# Peripheral blood iNKT cell activation correlates with liver damage during acute hepatitis C

**DOI:** 10.1172/jci.insight.155432

**Published:** 2022-01-25

**Authors:** Tina Senff, Christopher Menne, Christine Cosmovici, Lia Laura Lewis-Ximenez, Jasneet Aneja, Ruth Broering, Arthur Y. Kim, Astrid M. Westendorf, Ulf Dittmer, Norbert Scherbaum, Georg M. Lauer, Jörg Timm

**Affiliations:** 1 Institute of Virology, University Hospital Düsseldorf, Heinrich Heine University Düsseldorf, Medical Faculty, Düsseldorf, Germany.; 2 Laboratory of Viral Hepatitis, Oswaldo Cruz Institute, FIOCRUZ, Rio de Janeiro, Brazil.; 3 Gastrointestinal Unit, Massachusetts General Hospital and Harvard Medical School, Boston, Massachusetts, USA.; 4 Department of Gastroenterology and Hepatology, University Hospital Essen, University of Duisburg-Essen, Essen, Germany.; 5 Division of Infectious Diseases, Department of Medicine, Massachusetts General Hospital, Boston, Massachusetts, USA.; 6 Institute of Medical Microbiology and; 7 Institute for Virology, University Hospital Essen, University of Duisburg-Essen, Germany.; 8 LVR-Hospital Essen, Department of Addictive Behaviour and Addiction Medicine, Medical Faculty, University of Duisburg-Essen, Essen, Germany.

**Keywords:** Immunology, Virology, Hepatitis

## Abstract

Invariant NK T (iNKT) cells are implicated in viral clearance; however, their role in hepatitis C virus (HCV) infection remains controversial. Here, iNKT cells were studied during different stages of HCV infection. iNKT cells from patients with acute HCV infection and people who inject drugs (PWID) with chronic or spontaneously resolved HCV infection were characterized by flow cytometry. In a longitudinal analysis during acute HCV infection, frequencies of activated CD38^+^ iNKT cells reproducibly declined in spontaneously resolving patients, whereas they were persistently elevated in patients progressing to chronic infection. During the first year of infection, the frequency of activated CD38^+^ or CD69^+^ iNKT cells strongly correlated with alanine transaminase levels with particularly pronounced correlations in spontaneously resolving patients. Increased frequencies of activated iNKT cells in chronic HCV infection were confirmed in cross-sectional analyses of PWID with chronic or spontaneously resolved HCV infection; however, no apparent functional differences were observed with various stimulation protocols. Our data suggest that iNKT cells are activated during acute hepatitis C and that activation is sustained in chronic infection. The correlation between the frequency of activated iNKT cells and alanine transaminase may point toward a role of iNKT cells in liver damage.

## Graphical abstract

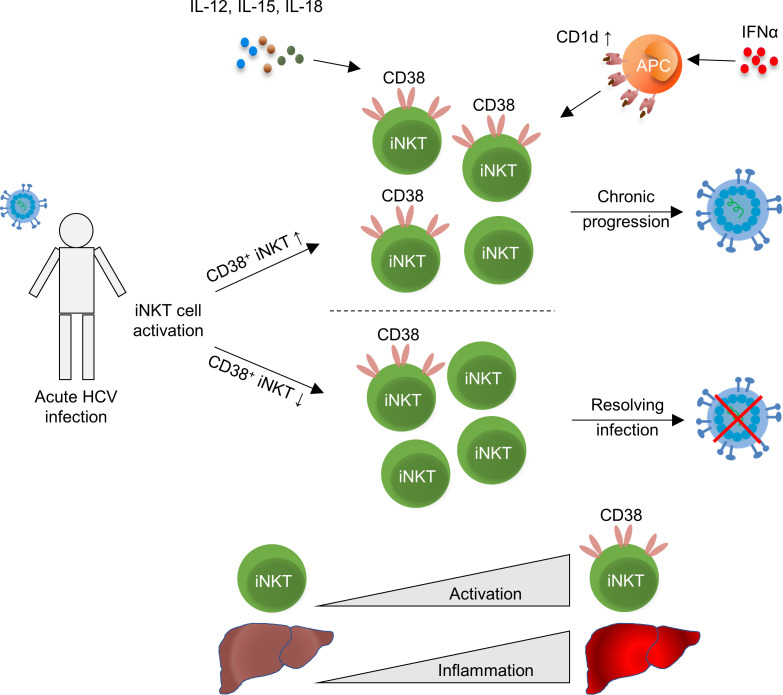

## Introduction

Even though highly effective direct-acting antivirals are available, hepatitis C virus (HCV) infection is still a major global healthcare problem, with 71 million chronically infected individuals worldwide. In people who inject drugs (PWID), there is a high prevalence of HCV infection, with up to 80% being seropositive for anti-HCV due to the common practice of sharing needles and other injection materials. Upon HCV exposure, the majority of patients with acute HCV infection remain asymptomatic or develop only mild and unspecific symptoms after an incubation period of 4–10 weeks. Approximately 60%–80% of individuals with acute hepatitis C develop chronic infection, defined as persistent viremia for more than 6 months after HCV exposure (1). The remaining individuals achieve immune control and spontaneously clear the infection (1, 2).

Previous work has elucidated the role of innate and adaptive immunity for HCV infection outcome. CD4^+^ and CD8^+^ T cell responses in particular have been repeatedly associated with viral clearance. Failure to achieve sufficient T cell responses due to functional CD8^+^ T cell exhaustion or substitutions within CD8^+^ T cell epitopes linked to CD8^+^ T cell escape are associated with viral persistence (3–5). In recent years, genetic association studies as well as analysis of the phenotype and function of NK cells in HCV-infected individuals have highlighted the importance of innate immune cells such as NK cells for viral clearance. Due to their early activation after exposure, they have been implicated in the resolution of acute infection. Additionally, they might protect from persistent infection at an early stage prior to seroconversion (6–9).

However, little is known about NK T (NKT) cells, a rare lymphocyte subset that bridges the innate and adaptive immune system. The focus of this study is invariant NKT (iNKT) cells, also known as type I or classical NKT cells, which express an invariant T cell receptor (TCR) comprising a Vα24Jα18 chain paired with a Vβ11 chain (10). In contrast to conventional T cells, which react upon presentation of peptide antigens, the iNKT cell TCR recognizes glycolipid antigens presented by the MHC class I–like molecule CD1d. iNKT cells can respond to microbially derived glycolipids (11) as well as the prototypic synthetic lipid antigen α‑galactosylceramide (αGalCer), which is a potent activator of iNKT cells and is derived from a marine sponge (10).

iNKT cells not only play an important role in innate host defense against bacterial and fungal infections (12, 13), but are also implicated in viral infections such as HIV (14) and HBV. In HBV infection, low frequencies of peripheral iNKT cells have been reported in patients with chronic hepatitis B that increase to normal levels upon viral clearance (15). However, in HCV infection, contradicting reports about iNKT cell frequencies exist, suggesting either decreased frequencies in chronic HCV infection or no association between frequency and HCV infection outcome (16–18). In humanized mouse hepatitis virus models, iNKT cells inhibit HCV replication by IFN-γ secretion (19). However, the mechanisms driving the activation of these cells in HCV infection remain to be elucidated.

Here, we analyzed iNKT cell frequencies and phenotype as well as a possible association with HCV infection outcome in 2 independent patient cohorts. iNKT cells were characterized longitudinally during acute HCV infection with different infection outcome and in a cohort of treatment-naive PWID, comprising anti-HCV seropositive PWID with and without detectable HCV RNA. Taken together, our data suggest an association between the phenotype of iNKT cells and HCV infection outcome as well as between iNKT cell phenotype and liver damage, and provide possible mechanisms for their in vivo activation. This expands the knowledge about this rare lymphocyte subset and highlights a functional role in HCV immunity.

## Results

### The activation phenotype of circulating iNKT cells associates with the outcome of HCV infection.

Unlike T and NK cells — their influence on the outcome of HCV infection has been analyzed in great detail (20, 21) — little is known about a possible relationship between the phenotype and functionality of iNKT cells and HCV control. In order to elucidate the role of iNKT cells at different stages of HCV infection, we performed flow cytometric analysis on PBMC samples from patients with acute HCV infection over a time course of up to 1 year after estimated time of infection (ETI).

**Figure 1:**
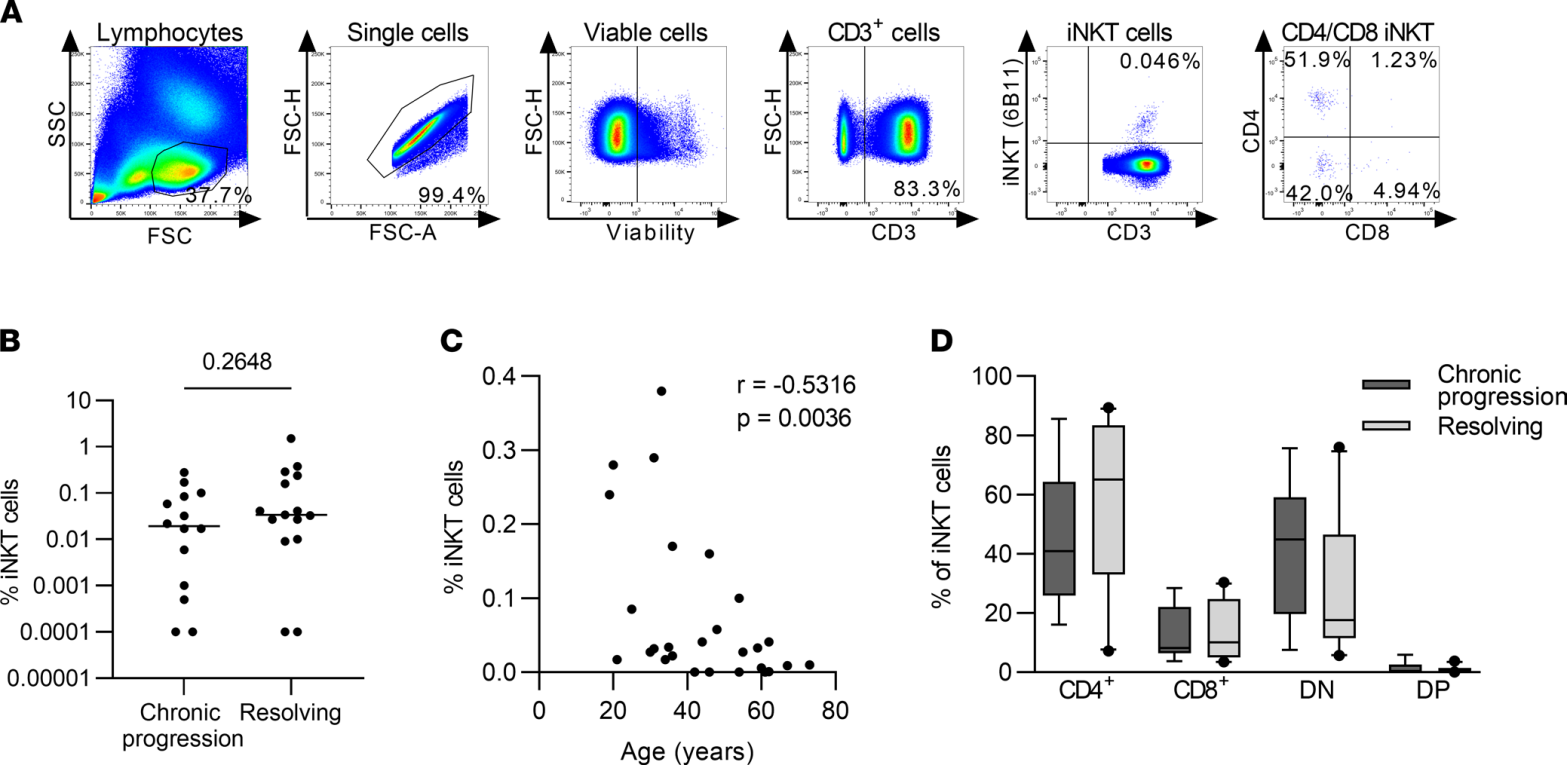
Frequencies of iNKT cells are stable during acute HCV infection. PBMC samples from the acute phase of HCV infection from chronic progressors (*n* = 15) and resolvers (*n* = 15) were analyzed by flow cytometry. (**A**) Representative gating strategy for Vα24Jα18^+^ invariant NK T (iNKT) cells. (**B**) The frequency of iNKT cells of all CD3^+^ T cells 12 weeks after estimated time of infection (ETI). Groups were compared by Mann-Whitney *U* test. (**C**) Association of iNKT cell frequency with the age in years of the corresponding donor at 12 weeks after ETI was calculated by Spearman’s correlation analysis. (**D**) iNKT cells were subgrouped according to the expression of CD4 and CD8 into CD4^+^, CD8^+^, double-negative (DN) and double-positive (DP) iNKT cells. Box plots depict the frequency of CD4^+^, CD8^+^, DN, and DP iNKT cells at 12 weeks after ETI. Samples with less than 20 iNKT cells were excluded from phenotypical analysis.

**Figure 2:**
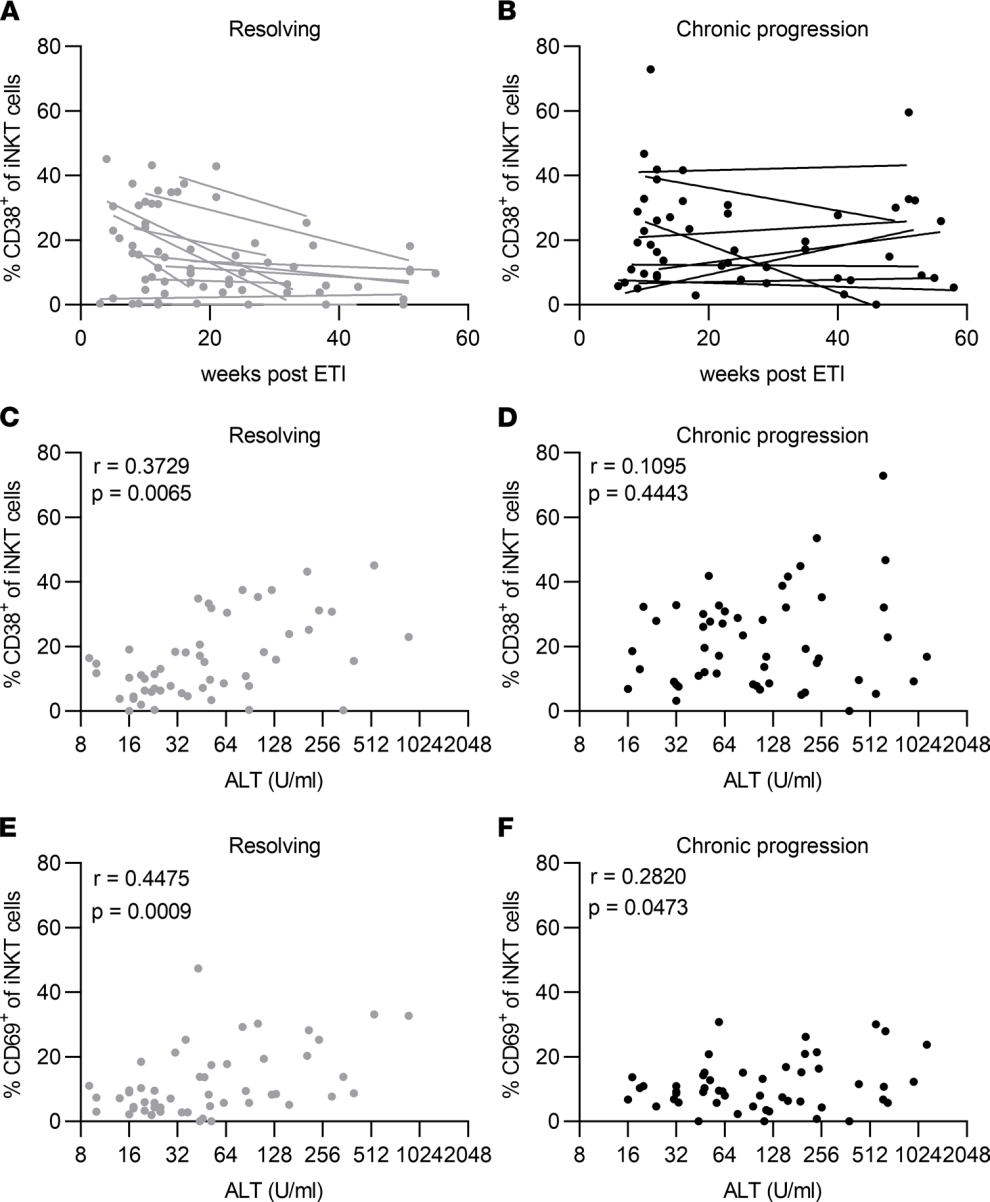
Chronic HCV progression associates with elevated activation of iNKT cells. PBMC samples from the acute phase of HCV infection from chronic progressors (*n* = 15) and resolvers (*n* = 15) were analyzed by flow cytometry. Samples with less than 20 iNKT cells were excluded from phenotypical analysis. (**A** and **B**) Percentage of CD38^+^ iNKT cells at indicated time points after ETI in resolvers (**A**) and progressors (**B**). Each dot represents an individual sample, and each line shows the overall trend for an individual patient over time calculated by linear regression analysis. Patients with more than 2 time points available were included in the analysis. (**C** and **D**) Correlation of alanine transaminase (ALT) levels with the frequency of CD38^+^ iNKT cells in all patients with known ALT level in the first year after ETI that either resolved (**C**) and progressed to chronic HCV (**D**). Pearson correlation analysis was used. (**E** and **F**) Correlation of ALT levels with the frequency of CD69^+^ iNKT cells in all patients with known ALT level in the first year after ETI that either resolved (**E**) or progressed to chronic HCV (**F**). Pearson correlation analysis was used.

First, we analyzed frequencies of iNKT cells using the gating strategy shown in Figure 1A. HCV progressors and resolvers showed similar frequencies of iNKT cells in peripheral blood in acute infection around 12 weeks after the ETI (Figure 1B). iNKT cell frequencies strongly varied among patients, ranging from undetectable to 1.50% (median, 0.0195% in progressors and 0.0340% in resolvers), and frequencies negatively correlated with patient age (Figure 1C, *r* = –0.53164, *P* = 0.0036), as previously reported (22, 23). Of note, the frequencies were highly stable over time and were unaffected by viral replication or liver inflammation (Supplemental Figure 1, A and B; supplemental material available online with this article; https://doi.org/10.1172/jci.insight.155432DS1). Because different effector mechanisms have been linked to iNKT cell subpopulations depending on their expression of CD4 or CD8 (24), we analyzed the distribution of iNKT cells into CD4^+^, double-negative (DN), or CD8^+^ subsets, again observing similar frequencies in progressors and resolvers (Figure 1D). Longitudinal analysis of iNKT cell activation determined by CD38 expression in individual patients revealed a highly reproducible trend for declining frequencies of CD38^+^ iNKT cells over time in patients that spontaneously resolved infection (Figure 2A, mean slope of linear regression, –0.432, with a 95% CI of –0.949 to –0.056). Conversely, patients that progressed to chronic HCV infection lacked this trend for declining frequencies and had uniformly high levels of CD38^+^ iNKT cells during the first year of infection (Figure 2B, mean slope of linear regression, –0.028, with a 95% CI of –0.355 to 0.263). In order to elucidate, whether this activation is iNKT cell specific or a general effect on lymphocytes due to increased inflammation during acute hepatitis, we compared the expression of CD38 and CD69 on iNKT cells, as well as on CD4^+^ and CD8^+^ T cells longitudinally in each individual patient (Supplemental Figure 2). Indeed, the longitudinal pattern of CD38 expression on iNKT cells and T cells strongly overlapped, despite some differences in expression frequencies. In contrast, the early activation marker CD69 was nearly absent on conventional CD4^+^ and CD8^+^ T cells, whereas it was expressed on a substantial proportion of iNKT cells. Interestingly, CD69 showed fluctuating expression levels in each patient over time, indicating iNKT cell activation in response to environmental signals.

We next analyzed the activation status of iNKT cells in the context of liver damage, which is defined by serum levels of alanine transaminase (ALT). Both the frequency of CD38^+^ iNKT cells as well as the frequency of CD69^+^ iNKT cells positively correlated with ALT levels. When all samples collected up to 1 year after ETI were included into the analysis, the correlation was particularly robust for HCV resolvers (Figure 2, C and E; *r* = 0.3729, *P* = 0.0065 for CD38, and *r* = 0.4475, *P* = 0.0009 for CD69) and was absent or less strong for HCV progressors (Figure 2, D and F). This correlation between iNKT cell activation and ALT levels was observed in individual resolvers as well while only a fraction of chronic progressors showed this correlation (Supplemental Figure 3, A and B). Moreover, cross-sectional analysis of defined time points during acute hepatitis C confirmed this association of iNKT cell activation levels with ALT (Supplemental Figure 4). Interestingly, the association of iNKT cell activation and viral load was less reproducible (Supplemental Figure 5).

### CD38^+^ iNKT cells are enriched in peripheral blood of chronically HCV-infected PWID.

**Figure 3:**
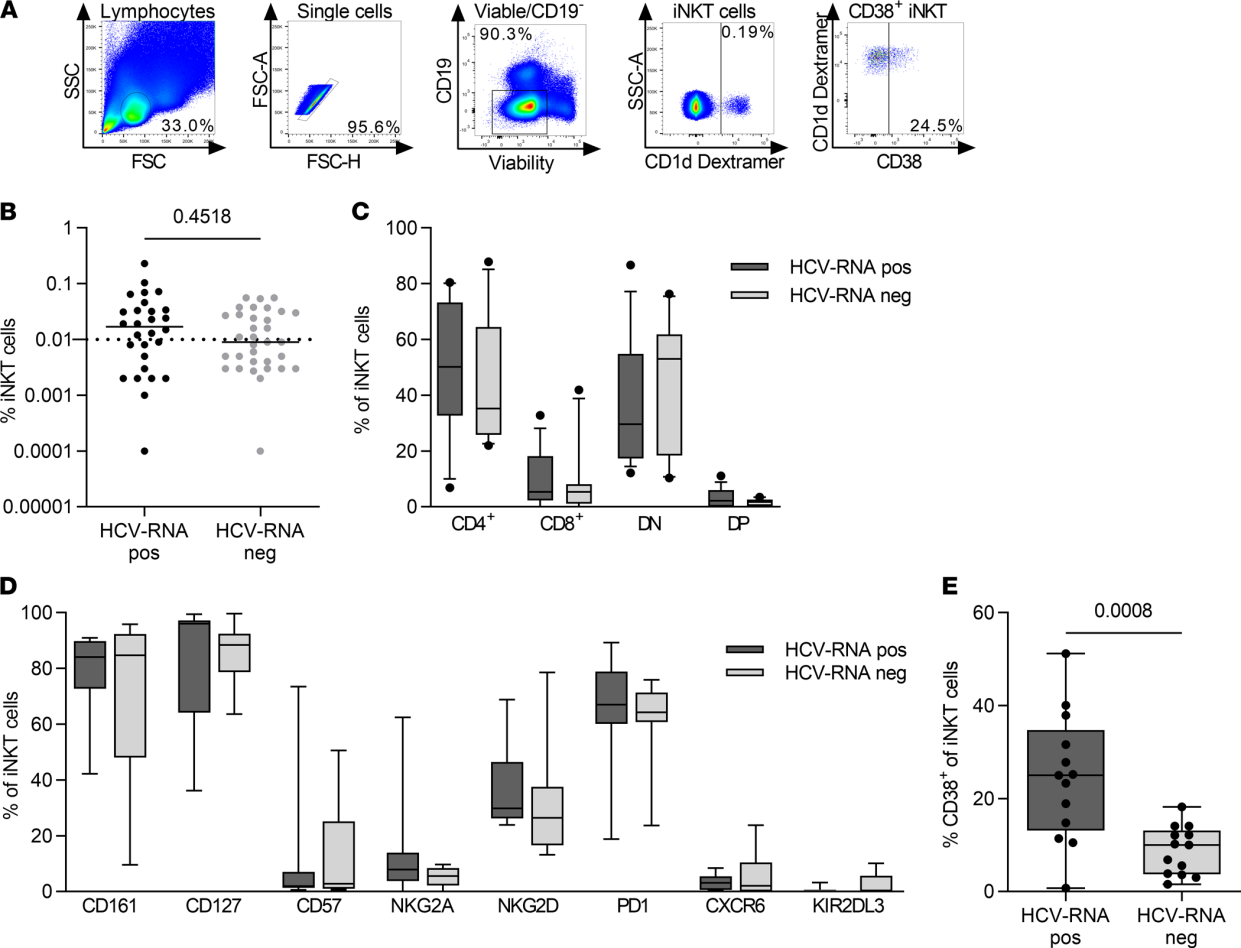
CD38^+^ iNKT cells are increased in HCV RNA–positive PWID compared with HCV RNA–negative PWID. PBMCs from HCV RNA–positive (*n* = 28) and HCV RNA–negative people who inject drugs (PWID) with detectable HCV antibodies (*n* = 33) were analyzed by flow cytometry. (**A**) Representative gating strategy for CD1d dextramer^+^ invariant NK T (iNKT) cells. (**B**) The frequency of iNKT cells of all CD19^–^ lymphocytes in HCV RNA–positive and HCV RNA–negative patients is shown. Groups were compared by Mann-Whitney *U* test. (**C**) iNKT cells were subgrouped according to the expression of CD4 and CD8 into CD4^+^, CD8^+^, double-negative (DN), and double-positive (DP) iNKT cells in HCV RNA–positive (*n* = 13) and HCV RNA–negative (*n* = 13) donors. (**D** and **E**) Percentage of iNKT cells positive for the indicated markers in HCV RNA–positive and –negative patients. Each bar represents results from 6–13 individual samples depending on sample availability and iNKT cell frequency. Groups were compared by unpaired *t* test. (**C–E**) Samples with iNKT frequency of less than 0.01% were excluded from phenotypical analysis. In box plots, horizontal bars indicate the medians, boxes indicate 25th to 75th percentiles, and whiskers indicate 10th and 90th percentiles.

In order to elucidate the phenotype of iNKT cells (Figure 3A) during later stages of HCV infection in more detail, a high-risk PWID cohort of anti-HCV–positive, treatment naive patients, with either chronic or spontaneously resolved HCV infection was studied. Frequencies of iNKT cells in HCV RNA–positive and HCV RNA–negative PWID ranged from undetectable to 0.23%. In line with results from the acute phase (Figure 1), frequencies and distribution of CD4^+^, DN, and CD8^+^ subsets were similar between both groups (Figure 3, B and C).

Analysis of multiple surface markers associated with T cell or NK cell differentiation and activation (Figure 3, D and E) showed higher levels of CD38^+^ iNKT cells in peripheral blood samples of HCV RNA–positive PWID (mean 24.5%) compared with HCV RNA–negative PWID (mean 8.9%, *P* = 0.0008), which was consistent with the results from the late phase of acute hepatitis C. No apparent differences were observed regarding the other markers included in the analysis (Figure 3D). Of note, in strong contrast to conventional T cells, virtually all iNKT cells showed expression of IL-7 receptor α chain (CD127), without any apparent difference between groups. Taken together, these data strongly indicate that chronic progression of HCV infection associates with an activated phenotype of iNKT cells.

### iNKT cells from HCV RNA–positive PWID are not functionally impaired.

**Figure 4:**
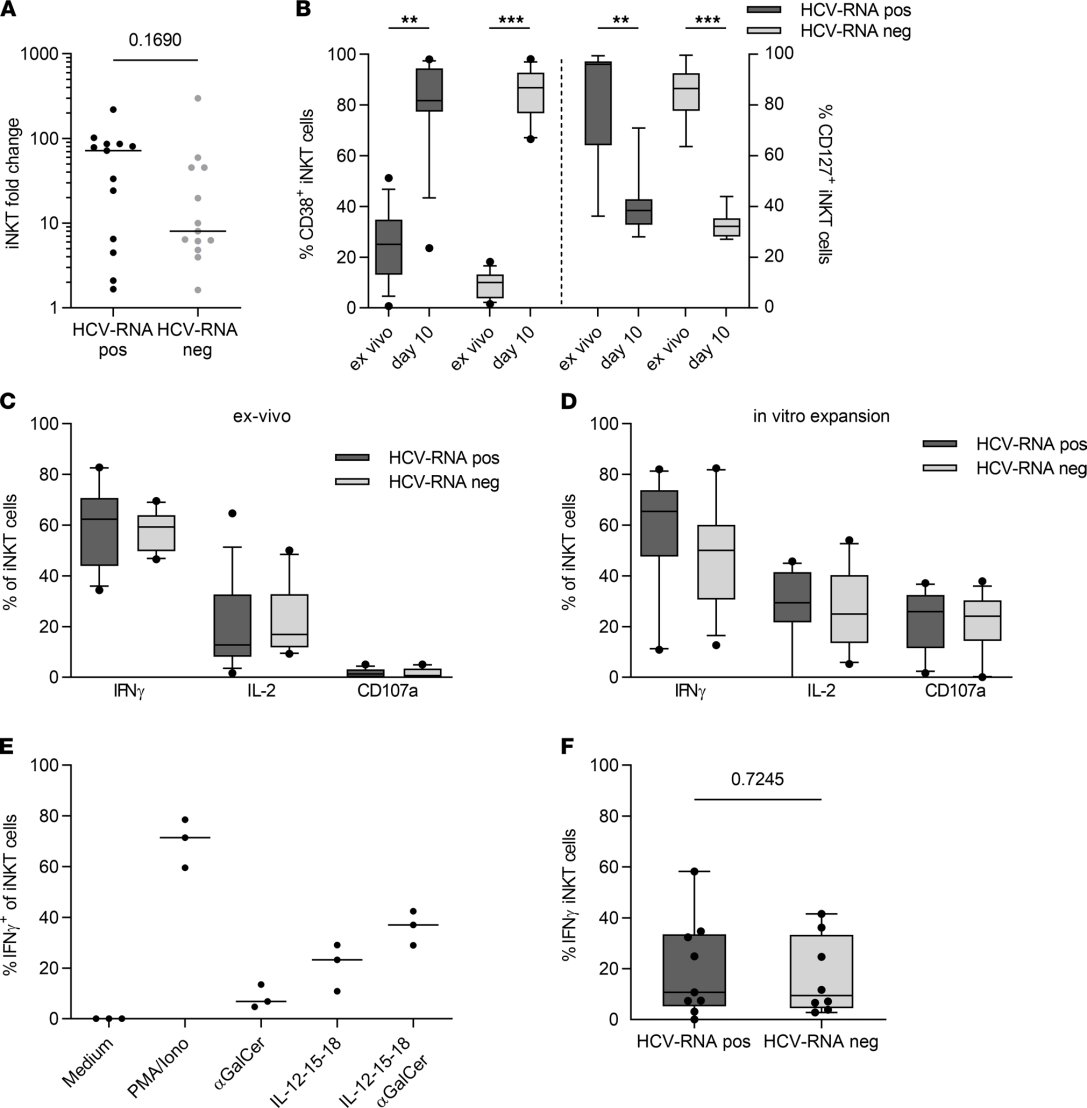
iNKT cell function does not differ between HCV RNA–positive and HCV RNA–negative PWID. (**A**) PBMCs from HCV RNA–positive (*n* = 13) and HCV RNA–negative (*n* = 13) donors were stimulated in vitro for 10 days with α-galactosylceramide (αGalCer) and IL-2, and fold change of invariant NK T (iNKT) cell frequency was analyzed. Groups were compared by Mann-Whitney *U* test. (**B**) Frequency of CD38^+^ and CD127^+^ iNKT cells in HCV RNA–positive and –negative patients ex vivo and after 10 days of in vitro expansion with αGalCer and IL-2 (1-way ANOVA, **P ≤ 0.01, ***P ≤ 0.001). (**C** and **D**) PBMCs from HCV RNA–positive (*n* = 13) and HCV RNA–negative (*n* = 13) donors were stimulated in vitro with phorbol myristate acetate (PMA) and ionomycin for 5 hours in presence of brefeldin A (BFA) for the last 4 hours ex vivo (**C**) or after 10 days (**D**) of expansion with αGalCer and IL-2. Intracellular cytokine staining was performed for IFN-γ and IL-2 and degranulation was analyzed by staining of CD107a. (**E**) PBMCs from healthy donors were stimulated with PMA and ionomycin; αGalCer; a cocktail of IL-12, IL-15, and IL-18; or a combination of αGalCer, IL-12, IL-15, and IL-18 for 24 hours, and IFN-γ secretion was analyzed by intracellular cytokine staining (ICS). (**F**) PBMCs from HCV RNA–positive (*n* = 9) and HCV RNA–negative (*n* = 8) donors were stimulated with a combination of αGalCer and IL-12, IL-15, and IL-18 for 24 hours, with addition of BFA for the last 4 hours, and IFN-γ secretion was analyzed by ICS. Groups were compared by unpaired *t* test. In box plots, horizontal bars indicate the medians, boxes indicate 25th to 75th percentiles, and whiskers indicate 10th and 90th percentiles.

Because our data suggest iNKT cell activation during chronic hepatitis C, we sought to analyze iNKT cell function in patients with chronic versus resolved HCV infection. Specific expansion of iNKT cells from total PBMCs in the presence of αGalCer for 10 days showed robust in vitro proliferation of iNKT cells, ranging from a 1.6- to 299-fold expansion. Expansion of iNKT cells from PBMCs of HCV RNA–positive individuals was more robust compared with HCV RNA–negative individuals; however, the difference was not statistically significant (Figure 4A). Activation and expansion of iNKT cells led to the expression of CD38 on the majority of all iNKT cells after 10 days (Figure 4B), recreating the phenotype we observed ex vivo. In line with that in T cells (25), expression of the IL-7 receptor α chain (CD127) on iNKT cells was downregulated after stimulation and declined from 82.75% (± 20.43%) and 85.81% (± 10.28%) to mean levels of 39.37% (± 10.82%) and 32.45% (± 4.78%) in HCV RNA–positive and –negative individuals, respectively (Figure 4B). Interestingly, this divergent expression of CD38 and CD127 after in vitro stimulation was reflected in an ex vivo analysis of PBMCs from patients in the early phase of acute HCV infection (Supplemental Figure 6). The functionality of iNKT cells was assessed by cytokine secretion assays after ex vivo phorbol myristate acetate/ionomycin (PMA/ionomycin) stimulation of PBMCs from HCV RNA–positive and HCV RNA–negative patients. Intracellular cytokine staining revealed a strong potential of iNKT cells to secrete IFN-γ and IL-2 but a weak degranulation capacity, as determined by CD107a expression, with all effector readouts being similar between outcome groups (Figure 4C and Supplemental Figure 7). We observed similar functional results after in vitro expansion with αGalCer, although iNKT cells showed more pronounced CD107a expression after expansion (Figure 4D). We also established a protocol in which PBMCs were stimulated for 24 hours with a cocktail of αGalCer and IL-12, IL-15, and IL-18 in order to use a more physiological iNKT cell stimulus. This iNKT cell–specific activation showed an additive effect of αGalCer and ILs on IFN-γ secretion, resulting in overall high levels of IFN-γ–positive iNKT cells, again with no apparent differences between cells from HCV RNA–positive and HCV RNA–negative PWID (Figure 4, E and F). Overall, the functional data did not reveal major differences in iNKT function related to the outcome of HCV infection.

### Proinflammatory cytokines may contribute to iNKT cell activation.

**Figure 5:**
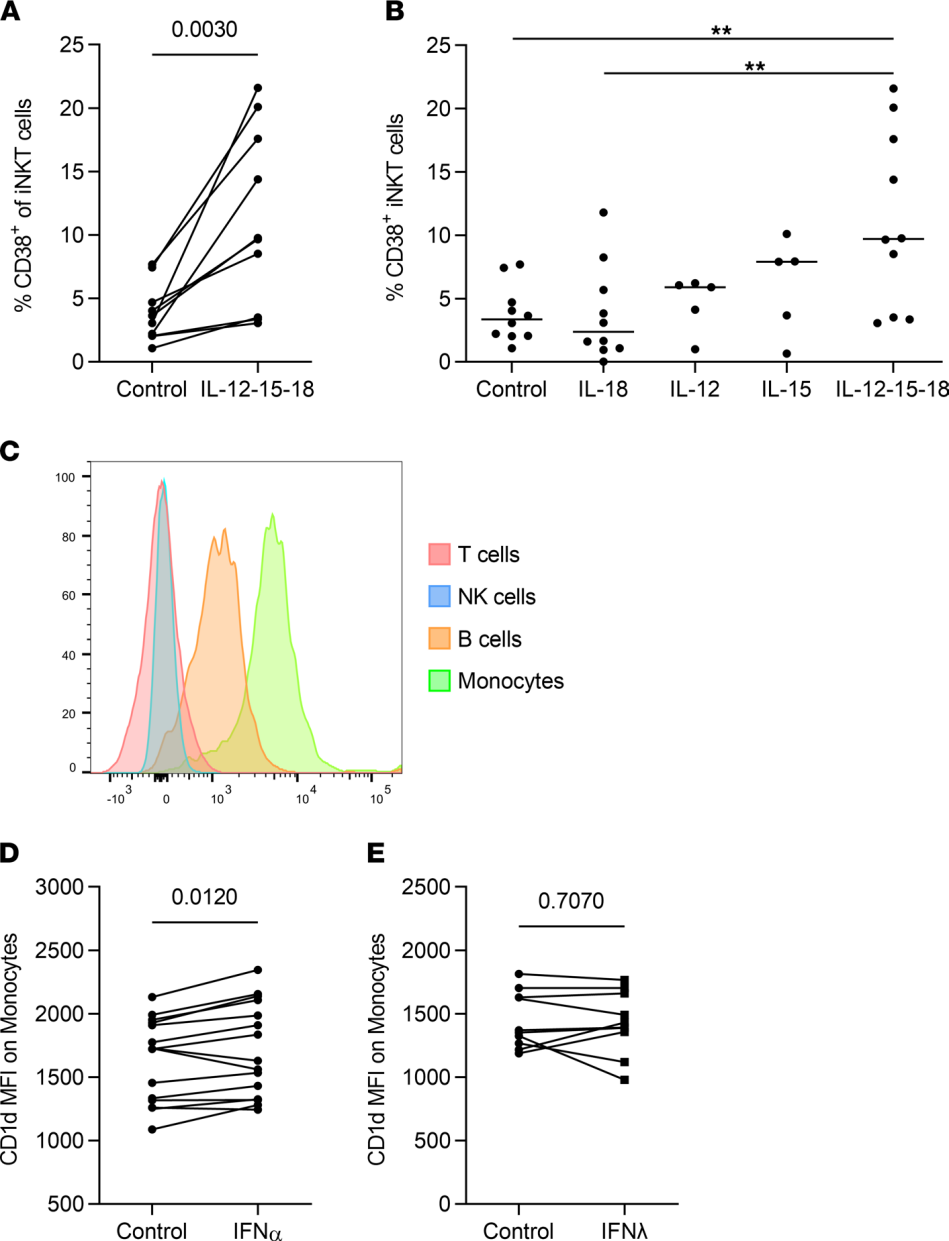
ILs enhance the expression of CD38 on iNKT cells, whereas type I IFNs induce an upregulation of CD1d on monocytes. (**A**) PBMCs from healthy donors were stimulated with IL-12, IL-15, and IL-18 for 24 hours, and the frequency of CD38^+^ iNKT cells was measured by flow cytometry (*n* = 10, paired *t* test). (**B**) Frequency of CD38^+^ iNKT cells after stimulation of PBMCs from healthy donors (*n* = 15) with IL-12, IL-15, and IL-18 or a combination of all 3 for 24 hours (1-way ANOVA, **P < 0.01). (**C**) CD1d mean fluorescence intensity (MFI) of various PBMC subsets was analyzed by flow cytometry. A representative experiment from 5 healthy donors is shown. (**D** and **E**) Healthy donor PBMCs were stimulated for 24 hours with 100 U/ml IFN-α2 or 1000 U/ml IFN-λ3 or were left untreated, followed by analysis of CD1d MFI of monocytes. Groups were compared by paired *t* test.

As our data suggested continued higher levels of activation for iNKT cells during chronic hepatitis C progression, we sought to determine possible mechanisms driving this iNKT activation. Therefore, PBMCs of healthy donors were stimulated with a combination of IL-12, IL-15, and IL-18, proinflammatory cytokines that are elevated in serum during chronic HCV infection (26–28), followed by analysis of activation markers on iNKT cells. Indeed, iNKT cells upregulated CD38, an effect that was mediated mainly by IL-12 and IL-15 in an additive manner (Figure 5, A and B). In order to analyze a possible TCR-mediated activation, we determined the expression of its ligand CD1d on various PBMC cell types. As expected, only professional antigen-presenting cells (APCs) in the peripheral blood, namely monocytes and to a lesser extent B cells, expressed CD1d (Figure 5C). Because high levels of type I IFNs and an IFN-induced gene signature are hallmarks of chronic HCV infection as well as because of the well-described role of type III IFNs in HCV clearance (20, 29, 30), the impact on iNKT cell activation in the context of CD1d expression on APCs was determined. Upon stimulation with type I IFN (IFN-α2a), there was a subtle but significant increase in the expression levels of CD1d, suggesting a potentially increased interaction of CD1d with the TCR of iNKT cells upon IFN-α signaling (Figure 5D, *P* = 0.012) that was not observed after stimulation with IFN-λ3 (Figure 5E). Notably, expansion of iNKT cells from monocyte-depleted PBMCs and, thus, partial depletion of CD1d from the culture resulted in reduced levels of CD38^+^ iNKT cells and reduced iNKT cell expansion (Supplemental Figure 8), outlining the importance of CD1d expression on professional APCs during iNKT cell activation.

### Intrahepatic iNKT cells have an activated phenotype compared with peripheral iNKT cells.

**Figure 6:**
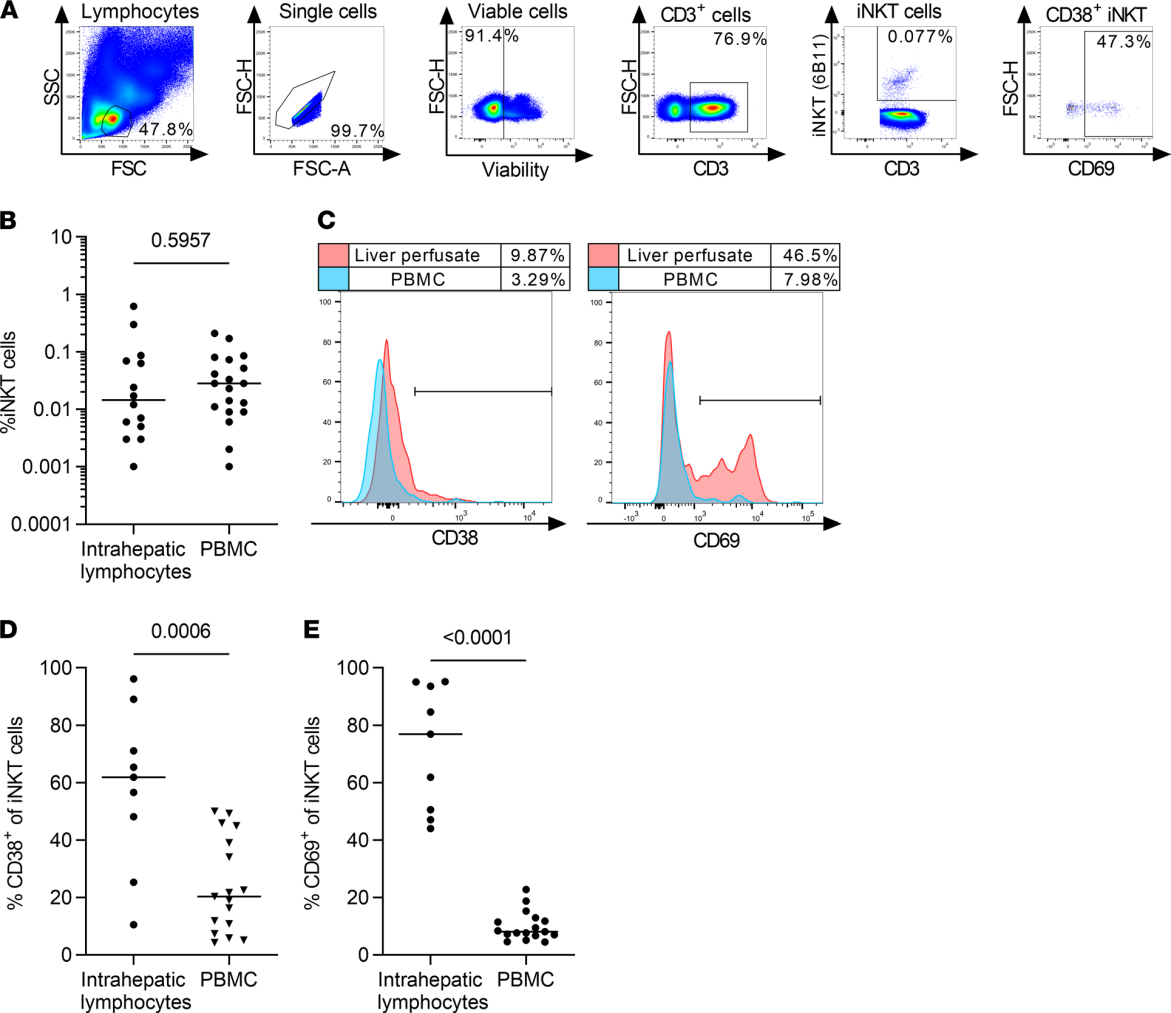
Intrahepatic iNKT cells are more activated than peripheral blood iNKT cells. (**A**) Representative gating strategy for Vα24Jα18^+^ iNKT cells from liver perfusates. (**B**) Frequency of intrahepatic and peripheral blood iNKT cells of all CD3^+^ T cells from nonmatched donors (liver perfusate, *n* = 14; PBMC, *n* = 19; Mann-Whitney *U* test). (**C**) Expression of CD38 and CD69 on iNKT cells was analyzed in liver perfusates and peripheral blood of matched donors. A representative experiment is shown from 2 independent donors. (**D** and **E**) The frequency of CD38^+^ and CD69^+^ iNKT cells in liver perfusates and PBMCs from nonmatched healthy donors was analyzed by flow cytometry (liver perfusate, *n* = 9; PBMC, *n* = 17; unpaired *t* test).

Because it has been shown that an activated phenotype is a common feature of intrahepatic T cells and not only a hallmark of HCV-specific T cells, we next determined the general activation status of iNKT cells in human livers (31). Mononuclear cells were isolated from perfusates of liver tissue resected during cancer surgery and analyzed by multicolor flow cytometry (Figure 6A). Lymphocytes from liver perfusates showed similar frequencies of iNKT cells compared with peripheral blood lymphocytes (Figure 6B, 0.015% and 0.028% median frequency). Interestingly, comparison of intrahepatic with peripheral blood iNKT cells either from the same donor (Figure 6C) or from an independent cohort of healthy donors revealed substantially higher frequencies of activated iNKT cells positive for CD38 (Figure 6D, *P* = 0.0006) as well as CD69 (Figure 6E, *P* < 0.0001) in the liver. This supports that iNKT cells with an activated phenotype are not only enriched in persistent infection, but also in the intrahepatic lymphocyte compartment.

## Discussion

Despite the well-described role of iNKT cells in viral infections in the murine system (32–35), iNKT cell studies of human viral infections are still rare due to experimental limitations and the extreme scarcity of this cell population in human peripheral blood. Therefore, studies in well-defined patient cohorts in the context of known viral infections are key to gaining further insights into human iNKT cell biology. In this study, we performed a detailed analysis of iNKT cells in a cohort with acute HCV infection as well as in an independent cohort with either chronic or spontaneously resolved HCV infection. The results show profound alterations in iNKT cell activation — marked by CD38 or CD69 expression — depending on the outcome of HCV infection. Enrichment of CD38^+^ iNKT cells in the peripheral blood during the transition phase from acute infection to chronic progression of HCV infection was sustained during the chronic stage. Interestingly, we also observed a strong correlation between the frequency of activated iNKT cells and ALT levels during acute HCV infection. Collectively, our data demonstrate that iNKT cells are activated during hepatitis C and that activation of iNKT cells associates with liver damage.

In the context of HCV infection, the majority of reports on iNKT cells focus on the analysis of their frequency (18, 36) and rarely describe analyses of the iNKT cell phenotype according to infection outcome (16). The current study characterizes the frequency and phenotype of iNKT cells in acutely infected patients with distinct infection outcomes and a high-risk cohort of anti-HCV–positive PWID. To our knowledge, this is the first comparative analysis of iNKT cells in acute, chronic, and spontaneously resolved HCV infection. In accordance with previous human iNKT cell studies, iNKT cell frequencies in the peripheral blood were highly variable between individuals (37, 38). In our study, the frequency of circulating iNKT cells was similar between groups and was not influenced by the HCV infection status. While previous studies by Inoue et al. (17) and van der Vliet et al. (18) could not detect an influence of HCV on iNKT cell frequency, consistent with our findings, other studies reported decreased iNKT cell frequencies in chronic HCV infection compared with controls (16). Previously described factors, such as age (22, 23) and sex (39), known to affect iNKT cell frequency might possibly be responsible for the discrepant results between studies. Of note, while iNKT cell frequencies did not differ between male and female patients, a negative correlation between age and iNKT cell frequency in the acute HCV cohort and in tendency in the PWID cohort (Supplemental Figure 9) was also observed in our study (22, 23).

The most striking finding regarding the iNKT cell phenotype in different infection outcomes was the downregulation of CD38 and CD69 in the context of HCV resolution. iNKT cell activation in the HCV setting has thus far been described only once in a cohort of exposed uninfected healthcare workers (40). In our study, longitudinal analysis of patients with acute HCV infection revealed a continuous and reproducible decrease of the activation status of iNKT cells in patients who resolved acute HCV infection, whereas patients who progressed to chronic HCV infection sustained high iNKT activation levels throughout the acute phase. Higher frequencies of activated iNKT cells in persistent infection were confirmed in a cross-sectional analysis of PWID with established chronic or spontaneously resolved HCV infection. Collectively, the existing data show an altered iNKT phenotype dependent on HCV infection outcome. If activated iNKT cells actively contribute to virus elimination or whether their activation declines after virus elimination and resolving inflammation requires further examination. Importantly when analyzing samples from individual patients, iNKT cells showed stronger and more variable CD69 expression compared with conventional T cells, indicating a faster response of these innate T cells to nonspecific activating stimuli.

Another key observation in this study is the correlation between the frequency of CD38- or CD69-positive iNKT cells and the ALT levels. This correlation was most robust in patients with spontaneously resolving HCV infection. Given the correlative nature of our study, we cannot distinguish whether iNKT cell activation contributes to hepatocyte killing or whether ALT elevation or the causal factors for liver damage also cause iNKT cell activation as part of the antiviral immune response. It has been well described that patients with acute hepatitis C, higher levels of ALT, and more severe symptoms have a higher likelihood for spontaneous resolution of infection (41–43). Although we cannot exclude that peripheral iNKT cell activation is a bystander of generalized immune activation due to liver inflammation, the stronger correlation in self-limited infection is intriguing and indicates that an early inflammatory response by activated iNKT cells may play a role in the outcome of HCV infection. In turn, failure of viral clearance during the acute phase results in sustained iNKT activation during the chronic phase. In support of this, several studies have shown direct cytotoxicity by iNKT cells (44) and an active contribution to αGalCer-mediated hepatitis (45, 46).

We also addressed the question of whether the observed iNKT cell activation in HCV-infected individuals can be caused by the cytokine milieu. Our data show that iNKT cells are directly activated by inflammatory cytokines, such as IL-12, IL-15, and IL-18, which are elevated during chronic HCV infection (26–28). In HCV-infected individuals, this is possibly accompanied by recognition of self-glycolipids presented by CD1d to the TCR of iNKT cells. In line with this, CD1d expression on monocytes was upregulated through IFN-α treatment — but not IFN-λ treatment— indicating a possible secondary activation mechanism by professional APCs at the site of inflammation elicited by sustained type I IFN signaling. Indeed, several studies (47–49) showed elevated CD1d expression on primary human hepatocytes as well as on liver-infiltrating APCs during chronic HCV infection. Hence, activation of peripheral iNKT cells during chronic HCV infection may be induced by IL-12 and IL-15 in the absence of an HCV-specific glycolipid and by continuous induction of IFN-stimulated genes with upregulation of CD1d.

Our analysis of intrahepatic human iNKT cells showed similar frequencies compared with the peripheral blood, with frequencies of iNKT cells in the human liver of less than 1%. This is in line with data from previous studies and in stark contrast to data from mice, in which iNKT cells can account for up to 30%–50% of all intrahepatic lymphocytes (49–51). Even though liver tissue was obtained from tumor resections from HCV-negative patients, in this study, data about frequency and phenotype of iNKT cells in human liver samples is valuable. It has been shown that intrahepatic T cells in general — not just HCV-specific T cells — are phenotypically distinct from peripheral blood T cells (31, 52). Lucas et al. (16) showed increased intrahepatic Vα24^+^Vβ11^+^ iNKT cell activation indicated by CD69 expression in a small number of patients. We provide further evidence for a strong iNKT cell activation phenotype in the liver, even in the absence of viral hepatitis, which confirms a distinct phenotype from circulating iNKT cells.

Whether the rise in activated iNKT cells in the periphery during acute and chronic HCV infection is caused directly by the virus or is a spillover effect from the highly activated intrahepatic iNKT cell compartment as a result from liver damage remains to be elucidated in future studies.

In summary, our study demonstrates that iNKT cells are activated during hepatitis C and that activation is sustained during chronic HCV infection. A strong correlation between the frequency of activated iNKT cells and ALT levels as a marker for liver damage might indicate a functional role for iNKT cells in the immune response against HCV and liver disease. Even though no apparent functional impairment of iNKT cells was observed during chronic hepatitis C, the observed iNKT cell phenotype can be indicative of involvement of this important immune cell subset during early HCV infection. Hence, additional studies are needed to address the exact role of iNKT cells in viral hepatitis in humans.

## Methods

### Study participants.

Whole blood samples from treatment-naive patients with a history of intravenous drug use were collected at the ward for inpatient detoxification treatment for people addicted to drugs or the clinic for opioid maintenance treatment at the Department for Addiction Medicine and Addictive Behavior of the LVR-Hospital Essen, Hospital of the University of Duisburg-Essen. Samples were tested by PCR for the presence of HCV RNA by commercial assays (Abbott RealTime HCV PCR assay or COBAS TaqMan HCV Test, v2.0), and the presence of anti-HCV antibodies was determined by a chemiluminescent microparticle immunoassay (ARCHITECT Anti-HCV from Abbott). According to their anti-HCV and HCV RNA status, PWID were divided into 2 groups: anti-HCV–seropositive patients with detectable HCV RNA (HCV RNA positive) and anti-HCV–seropositive individuals without detectable HCV RNA (HCV RNA negative). The patient characteristics of the PWID cohort are summarized in Table 1.

In addition, peripheral blood samples from individuals with acute HCV infection from North America and Brazil covering various transmission routes were collected during the first year after infection The ETI was either calculated according to peak ALT levels (ETI assumed to be 7 weeks before peak) or based on patient exposure history. Individuals were sampled multiple times over a period of 52 weeks after HCV exposure. According to their disease status at 12 months after ETI, individuals were grouped into ‘‘progressors’’ that developed chronic HCV viremia and ‘‘resolvers’’ that spontaneously cleared HCV without any treatment. Patient characteristics of the acute HCV cohort are shown in Table 2.

Additionally, PBMCs from healthy donors were processed from buffy coats collected from the center for blood donation from the University Hospital Düsseldorf and analyzed.

Intrahepatic lymphocytes were prepared from liver specimens obtained from fresh tumor resections of uninfected individuals (*n* = 14). The liver specimens were placed in a petri dish, and a cannula was positioned in an accessible hepatic vessel. The cannula was fixed, and remaining vessels were sealed using Histoacryl (Braun). The liver tissues were rinsed under light pressure with perfusion solution (Ca_2_^+^- and Mg_2_^+^-free Hank’s balanced salt solution, Biochrome) supplemented with 0.02 mg/ml gentamicin (Biochrome) and 20 mM HEPES (Biochrome) prewarmed to 37°C for 10 to 20 minutes at a flow rate of 30–40 ml/minute until nearly all the blood had been flushed out.

### Preparation of mononuclear cells.

PBMCs were isolated from EDTA anticoagulated whole blood or buffy coats from healthy donors and intrahepatic lymphocytes were isolated from the collected perfusate via Ficoll density centrifugation (Biochrome) and cryopreserved in 90% FBS (Biochrome) and 10% dimethyl sulfoxide (Roth).

### Frequency and phenotype analysis of iNKT cells.

Cryopreserved PBMCs were thawed and stained for iNKT cells with either APC-labeled αGalCer–loaded CD1d dextramer (Immudex) for 20 minutes at room temperature in the dark or with Vα24Jα18 antibody (clone 6B11) labeled with BV421 or PE for 15 minutes at 4°C in the dark. The dextramer and the Vα24Jα18 showed highly comparable iNKT cell staining patterns, a finding which was additionally validated by costaining with Vα24 and Vβ11 antibody (Supplemental Figure 10). Because the overlap of the cells positive for dextramer and Vα24Jα18 is not 100% (53, 54), all analyses within each cohort were done with the same reagent, and no comparisons between cohorts were conducted. Prior to fixation, cell surface staining was performed at 4°C for 20 minutes. Subsequently, iNKT cells were analyzed with BD FACSCanto II, BD LSRFortessa, or BD LSR II and analyzed with FlowJo 10.0.7 software. Gates were set according to fluorescence minus one controls. At least 500,000 events were recorded, and viability was over 90% for all analyzed samples. Samples with less than 20 or 0.01% iNKT cells were excluded from phenotypical analysis, as stated in the figure legends. All antibodies used are listed in Supplemental Table 1.

### Assessment of the expansion capacity of iNKT cells.

PBMCs were cultured for 10 days in RPMI Medium 1640 (Gibco, Thermo Fisher Scientific) supplemented with 10% FBS (Biochrome), 100 U/ml penicillin (Gibco), 100 μg/ml streptomycin (Gibco), 10 mM HEPES buffer (Gibco) (hereafter referred to as R10) supplemented with 25 IU/mL recombinant human IL‑2 (Roche, Merck) and 1 μg/mL αGalCer (Funakoshi). After 5–7 days, half of the total volume of R10 containing 25 IU/mL recombinant human IL‑2 was added to the culture. iNKT cell expansion and activation was evaluated via surface staining as described above.

### Functional analysis of iNKT cells.

PBMCs were analyzed ex vivo or after expansion as described above. PBMCs were stimulated with 1 μg/mL ionomycin (Calbiochem, Merck) and 10 ng/mL PMA (MilliporeSigma, Merck) for 5 hours in the presence of brefeldin A (BFA) (100 ng/mL, MilliporeSigma) and anti-CD107a antibody or 24 hours with αGalCer (1 μg/ml, Reprokine) and IL-12 (10 ng/ml, Reprokine), IL-15 (100 ng/ml), and IL-18 (50 ng/ml, Reprokine) with addition of BFA (100 ng/ml) for the last 4 hours of cultivation. PBMCs were surface stained and fixed with IC fixation buffer prior to permeabilization with 1× Permeabilization Buffer (both from eBioscience) and intracellularly stained with anti-human IFN-γ and anti-human IL-2 antibody.

### Stimulation of iNKT cells with IL-12, IL-15, and IL-18.

PBMCs were incubated with or without human IL-12 (10 ng/mL), IL-15 (100 ng/mL), and IL-18 (100 ng/mL) alone or in combination (10 ng/mL, 100 ng/mL, 50 ng/mL) over 24 hours, followed by staining of cell surface markers as described above.

### CD1d expression on peripheral blood cells.

CD1d expression on the cell surface of peripheral cells, including T cells, B cells, NK cells, and monocytes from healthy individuals was analyzed by flow cytometry. Monocytes were identified through FSC and SSC gating and expression of CD14, while T (CD3^+^), B (CD19^+^), and NK (CD3^–^/CD14^–^/CD19^–^ and CD56^+^) cells reside in a lymphocyte gate comprising cells with less granularity and size. Furthermore, peripheral blood cells were treated with or without 100 IU/ml IFN-α2a or 1000 IU/ml IFN-λ3 (both PBL Assay Science) over 24 hours, and CD1d expression was assessed on monocytes defined through CD14 and CD16.

### Statistics.

Statistical analyses of iNKT cell frequencies and function were performed using GraphPad Prism software. A *P* value less than or equal to 0.05 was considered statistically significant. Data were examined for normal distribution using the Shapiro-Wilk test, followed by a Grubbs’ test for equivalent outliers. For the comparison of 2 groups either a parametric or a nonparametric 2-tailed *t* test was performed. Three or more groups were compared by 1-way ANOVA or a Kruskal-Wallis test. Correlations were calculated with the parametric Pearson’s or nonparametric Spearman’s test. Trends in longitudinal data were analyzed by simple linear regression.

### Study approval.

This study was approved by the local ethics committees of the Medical Faculty of the University of Duisburg-Essen and the University Hospital Düsseldorf as well as the Partners Healthcare Human Research Committee (Boston, Massachusetts, USA) and the IRB of the Oswaldo Cruz Institute. All participants provided written documentation of informed consent.

## Author contributions

TS, CM, AMW, UD, GML, and JT conceptualized and designed the study and the experiments. TS, CM, CC, AMW, and JT conducted the experiments and analyzed and visualized the data. LLLX, RB, AYK, NS, GML, and JT acquired and prepared patients samples and clinical data. JA provided patient samples and was also responsible for the biobank where patient samples are collected. TS, CM, GML, and JT wrote the original manuscript. UD and JT supervised the study. JT acquired funding. All authors critically reviewed and edited the final manuscript.

## Supplementary Material

Supplemental data

## Figures and Tables

**Table 2:**
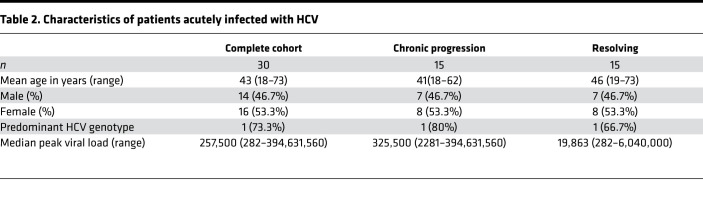
Characteristics of patients acutely infected with HCV

**Table 1:**
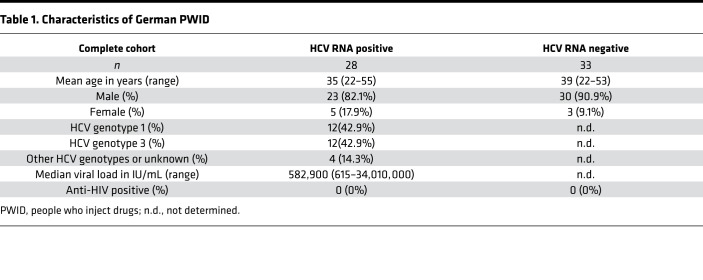
Characteristics of German PWID

## Version 1 12/14/2021

In-Press Preview

## Version 2 01/25/2022

Electronic publication
