# Supplementary material for: Peripheral blood iNKT cell activation correlates with liver damage during acute hepatitis C
Source: JCI Insight. 2022 Jan 25;7(2):e155432. doi: 10.1172/jci.insight.155432 (PMC8855829; doi:10.1172/jci.insight.155432)
Supplement: Supplemental data [file jciinsight-7-155432-s072.pdf]

Supplementary data

Invariant Natural Killer T cells with an activated phenotype correlate with liver damage during acute hepatitis C

Tina Senff[#], Christopher Menne[#], Christine Cosmovici, Lia L. Lewis-Ximenez, Jasneet Aneja, Ruth Broering, Arthur Y. Kim, Astrid Westendorf, Ulf Dittmer, Norbert Scherbaum, Georg M. Lauer, Jörg Timm

Table of contents

Supplementary Table 1

Supplementary Fig. 1

Supplementary Fig. 2

Supplementary Fig. 3

Supplementary Fig. 4

Supplementary Fig. 5

Supplementary Fig. 6

Supplementary Fig. 7

Supplementary Fig. 8

Supplementary Fig. 9

Supplementary Fig. 10

Supplementary Table 1. Antibodies used in this work.

Name	Supplier	Cat no.	Clone no.
αGalCer loaded CD1d dextramer APC	Immudex (Virum Denmark)	XD8002	n.a.
CD1d PerCPeFluor™710	eBioscience (Thermo Fisher Scientific, Waltham, MA)	46-0016-42	51.1
CD38 PerCP eFluor 710	eBioscience	46-0388-42	HB7
PE-Cy™7 Mouse Anti-Human CD127	BD Biosciences (San Jose, CA)	560822	HIL-7R-M21
CD161 PE-Cyanine7	eBioscience	25-1619-42	HP-3G10
CD57 FITC	eBioscience	11-0577-42	TB01
CD159a/NKG2A PC7	Beckman Coulter (Brea, CA)	B10246	Z199
Brilliant Violet 421™ anti-human CD279 PD-1	Biolegend (San Diego, CA)	329920	EH12.2H7
Fixable ViabilityDye eFluor® 506	eBioscience	65-0866	
CD19 APC-eFluor® 780	eBioscience	47-0199-42	HIB19
PE-Cy™7 Mouse Anti-Human CD107a	BD Biosciences	561348	H4A3
Anti-Human IFN gamma FITC	eBioscience	11-7319	4S.B3
IL2 PerCP-eFluor 710	eBioscience	46-7029-42	MQ1-17H12
CD4 PE	eBioscience	12-0049-42	RPA-T4
CD8a eFluor® 450	eBioscience	48-0088-42	RPA-T8
BUV395 Mouse Anti-Human CD8	BD Biosciences	563795	RPA-T8
BUV737 Mouse Anti-Human CD4	BD Biosciences	564306	SK3
Brilliant Violet 421™ anti-human TCR Vα24-Jα18 (iNKT cell)	Biolegend	342916	6B11
FITC anti-human CD69	Biolegend	310903	FN50
PE/Dazzle™ 594 anti-human CD3	Biolegend	300336	HIT3a
PE/Cy7 anti-human CD38	Biolegend	356608	HB-7
LIVE/DEAD™ Fixable Near-IR Dead Cell Stain Kit	Invitrogen (Thermo Fisher Scientific, Waltham, MA)	L34976	n.a.
BV786 Mouse Anti-Human CD4	BD Biosciences	740962	RPA-T4
CD38 PE-Cyanine7	eBioscience	25-0389-42	HIT2

APC anti-human CD69	Biolegend	310910	FN50
CD3 PerCP-Cyanine5.5	eBioscience	45-0037-42	OKT3
PE anti-human CD127 (IL-7Rα)	Biolegend	351304	A019D5

A

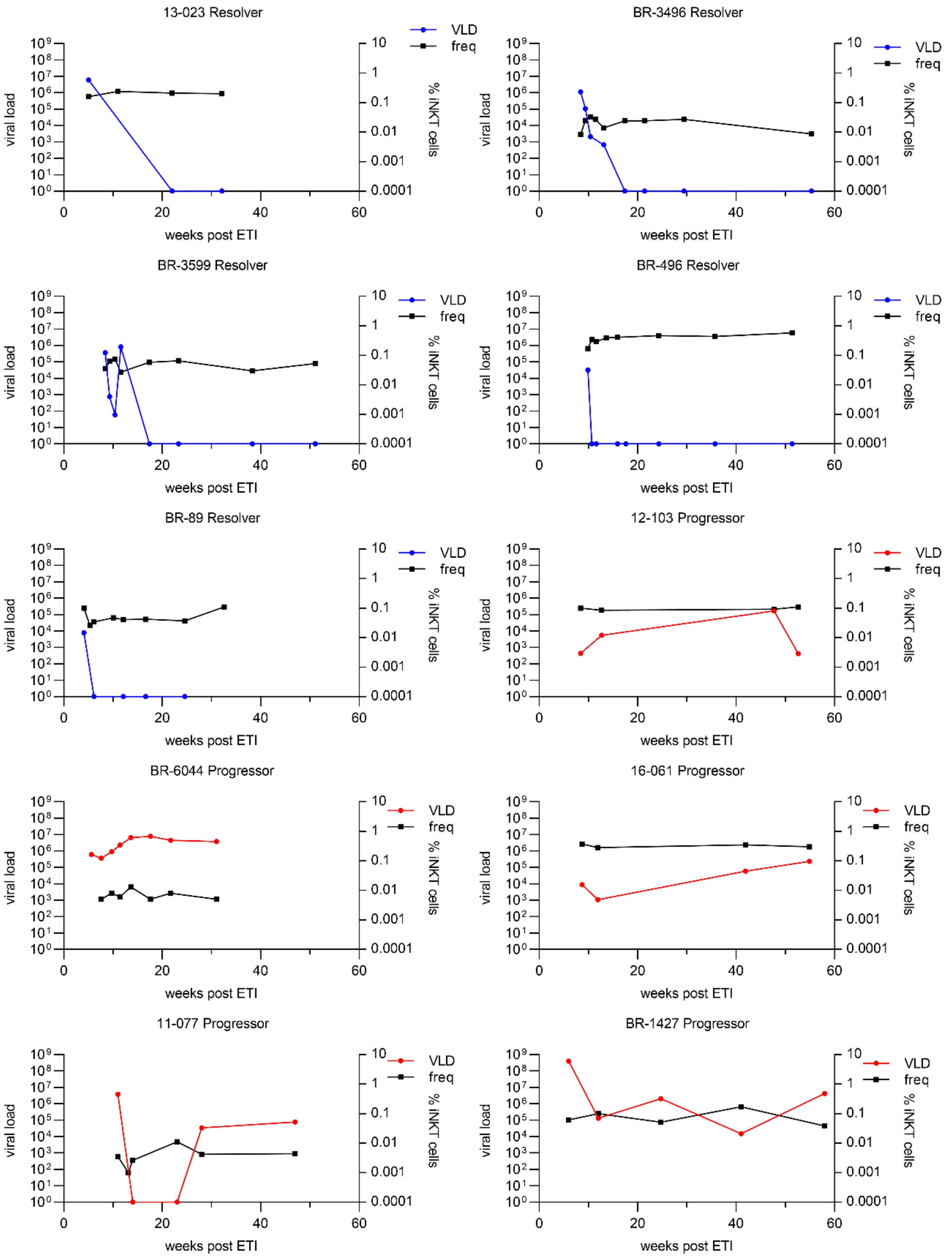
13-023 Resolver
BR-3496 Resolver
BR-3599 Resolver
BR-496 Resolver
BR-89 Resolver
12-103 Progressor
BR-6044 Progressor
16-061 Progressor
11-077 Progressor
BR-1427 Progressor
viral load
% iNKT cells
weeks post ETI
VLD
freq
10^0 10^1 10^2 10^3 10^4 10^5 10^6 10^7 10^8 10^9
0.0001 0.001 0.01 0.1 1 10
0 20 40 60

B

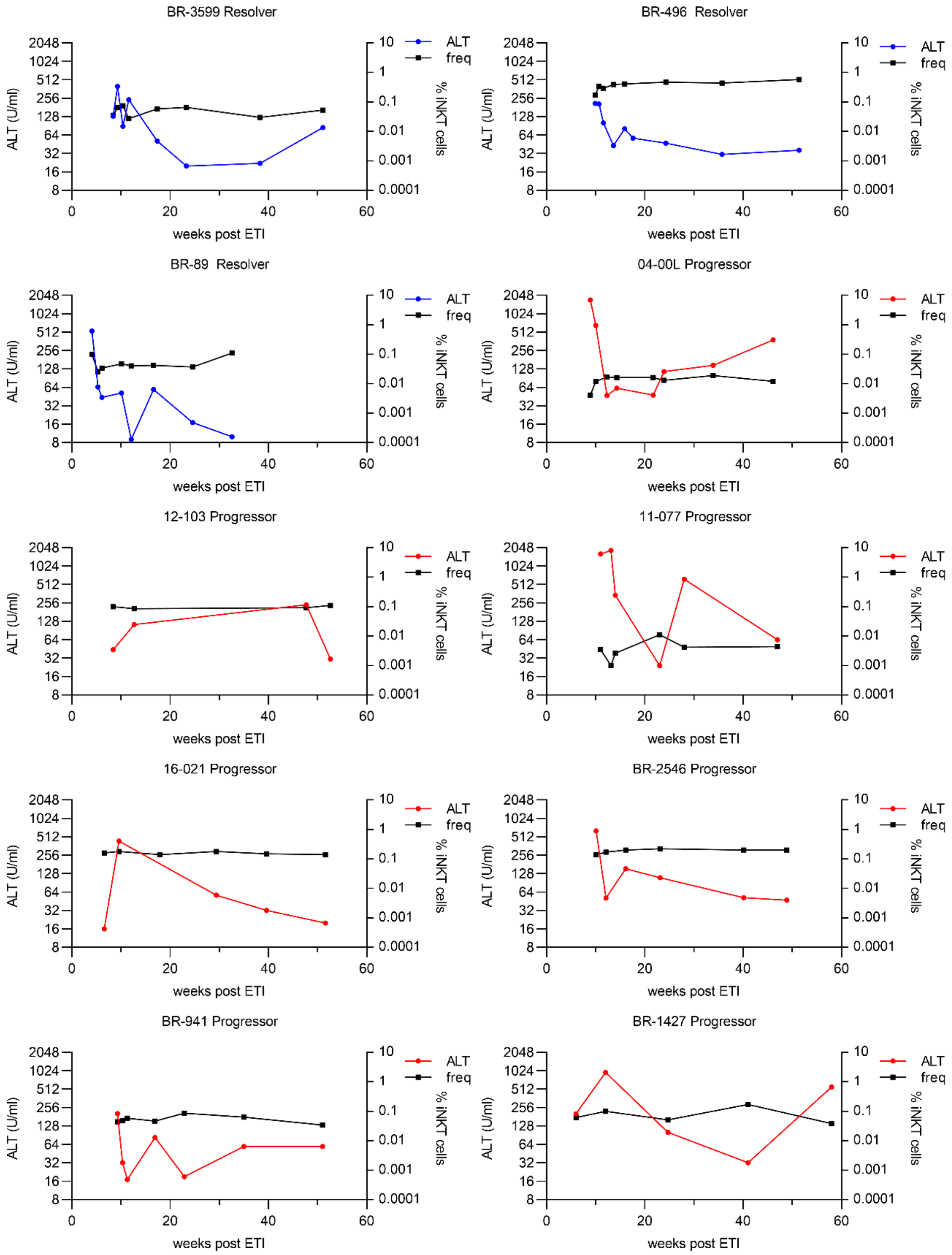

Supplementary Fig. 1. Invariant NKT cell frequency is unaltered by viral replication or serum ALT levels. Frequency of iNKT cells during the first year post ETI is depicted in black squares. Viral load (A) and ALT (B) is depicted by blue circles

for resolvers and red circles for chronic progressors. Exemplary patients that had the highest number of longitudinal samples with available clinical parameters are shown.

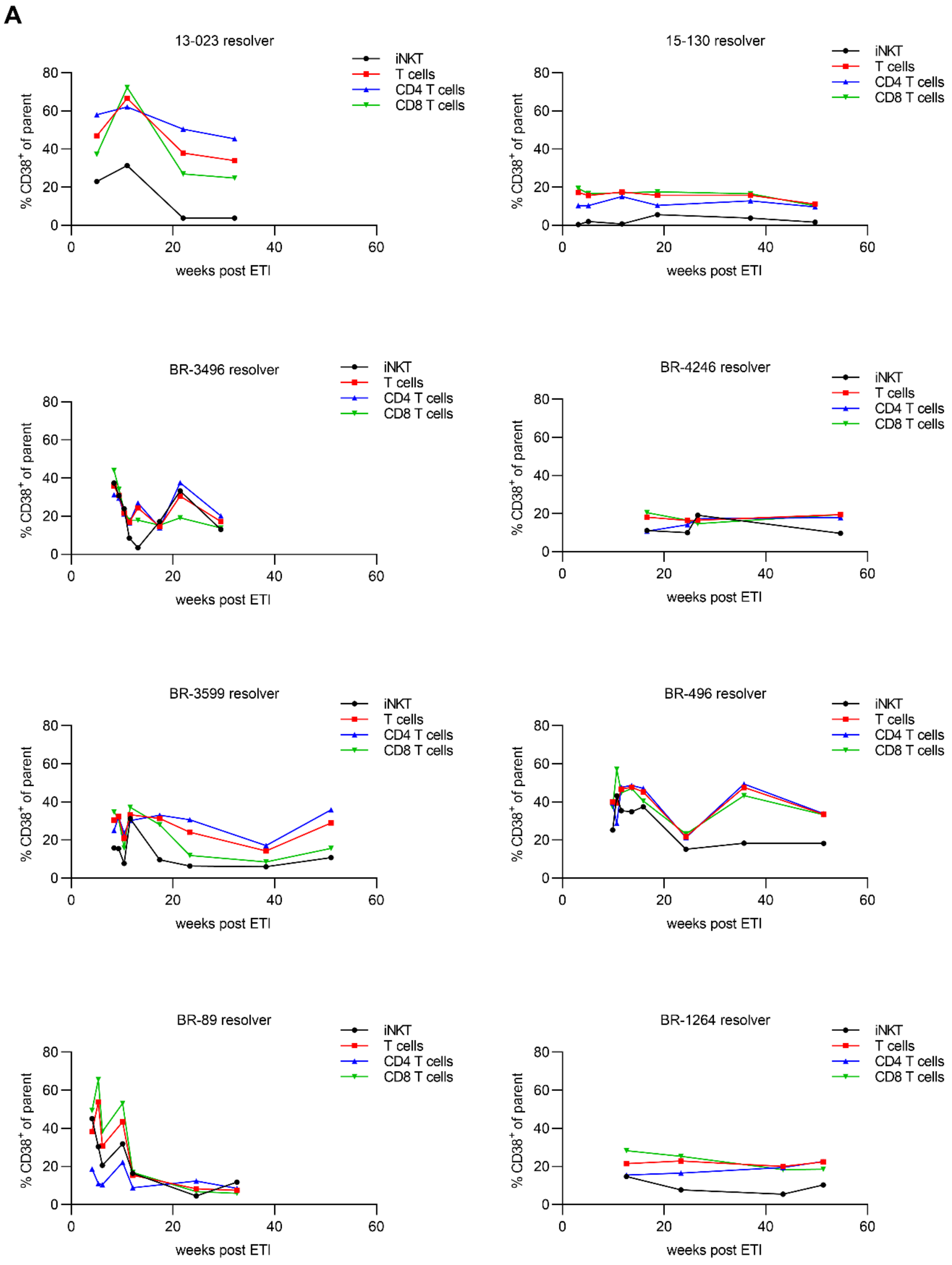
A
13-023 resolver
15-130 resolver
BR-3496 resolver
BR-4246 resolver
BR-3599 resolver
BR-496 resolver
BR-89 resolver
BR-1264 resolver
iNKT
T cells
CD4 T cells
CD8 T cells
% CD38+ of parent
weeks post ETI
0
20
40
60
80

B

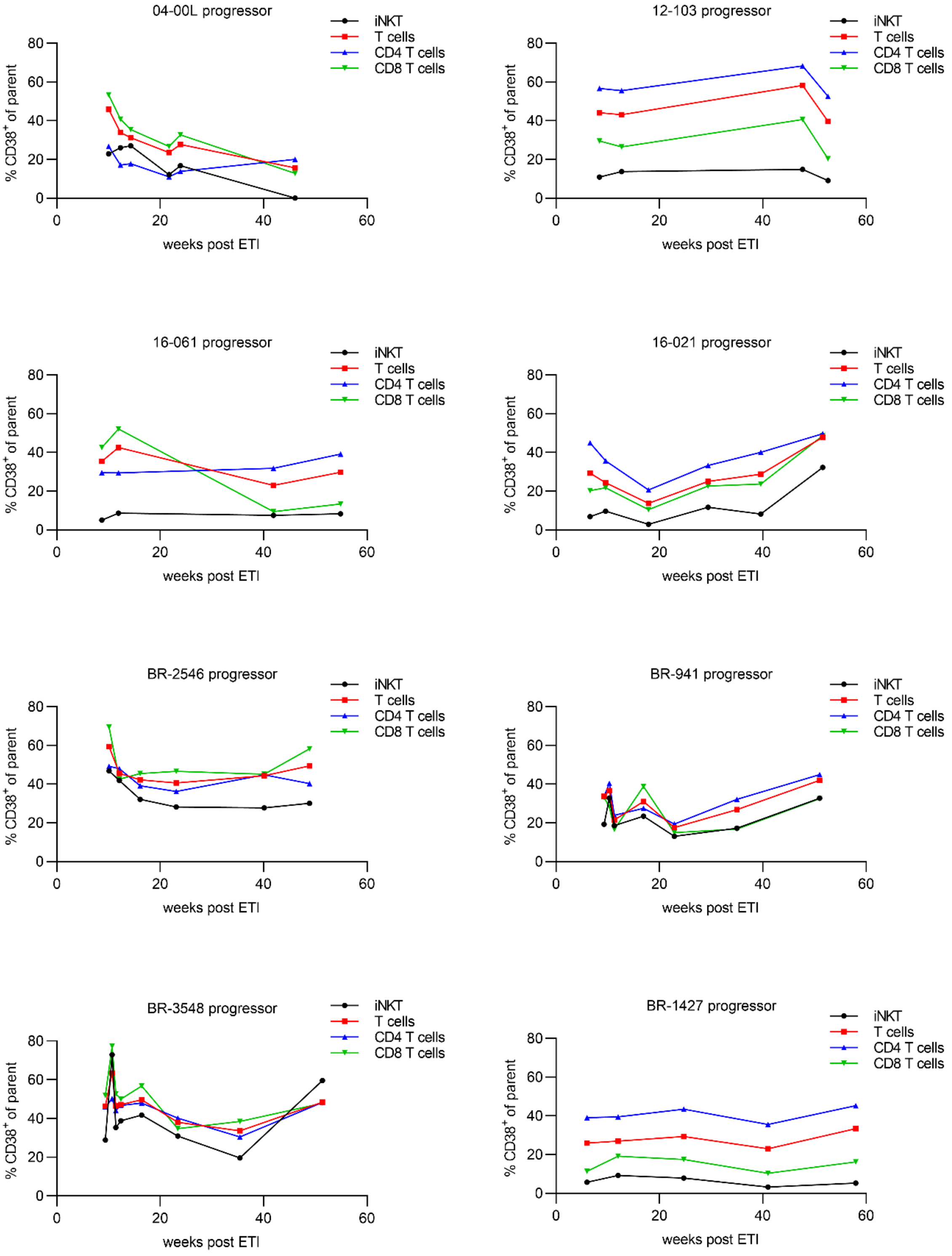
04-00L progressor
12-103 progressor
16-061 progressor
16-021 progressor
BR-2546 progressor
BR-941 progressor
BR-3548 progressor
BR-1427 progressor
iNKT
T cells
CD4 T cells
CD8 T cells
% CD38+ of parent
weeks post ETI
0
20
40
60
80

C

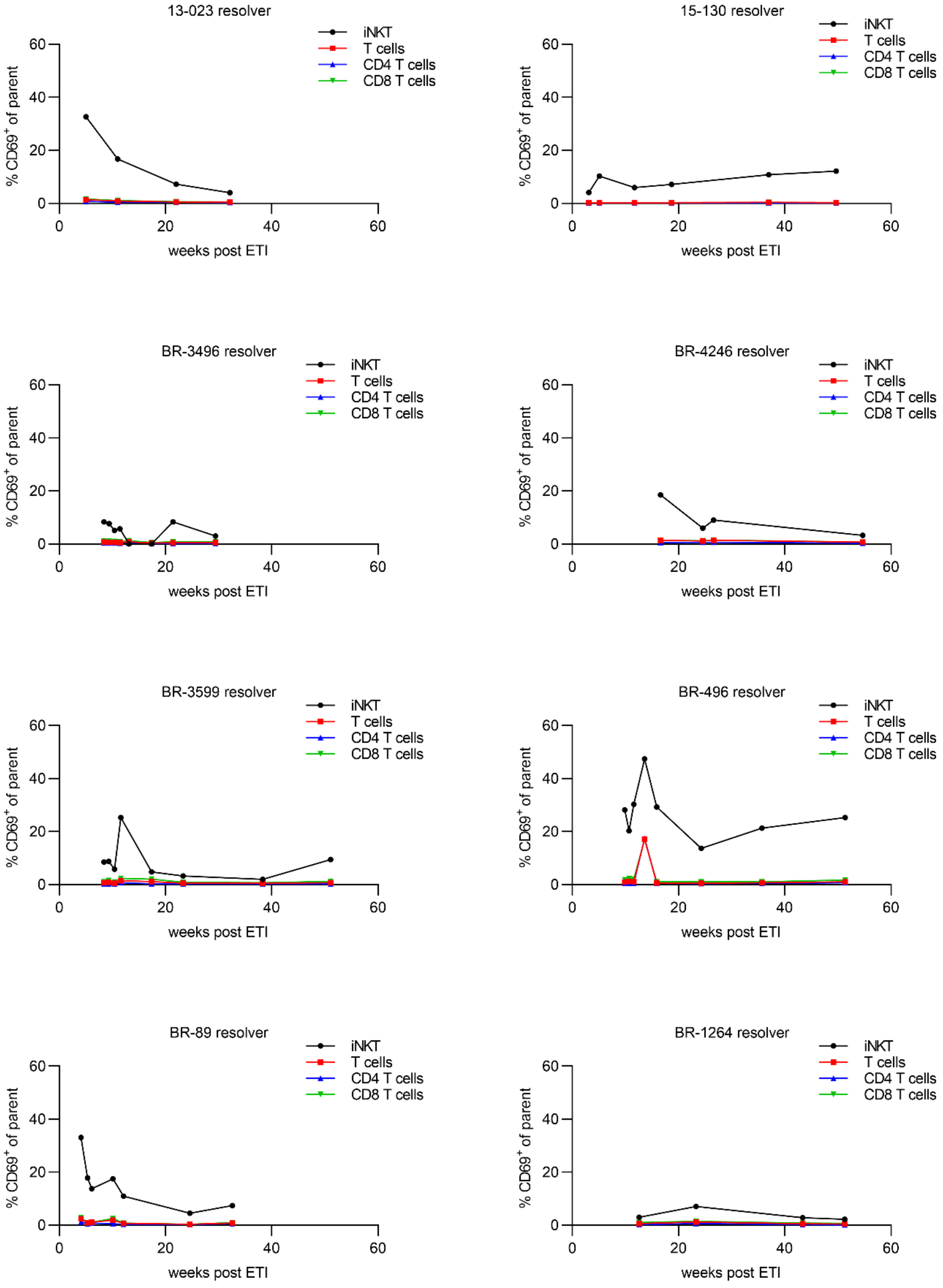
13-023 resolver
15-130 resolver
BR-3496 resolver
BR-4246 resolver
BR-3599 resolver
BR-496 resolver
BR-89 resolver
BR-1264 resolver
iNKT
T cells
CD4 T cells
CD8 T cells
% CD69^{+} of parent
weeks post ETI
0
20
40
60

D

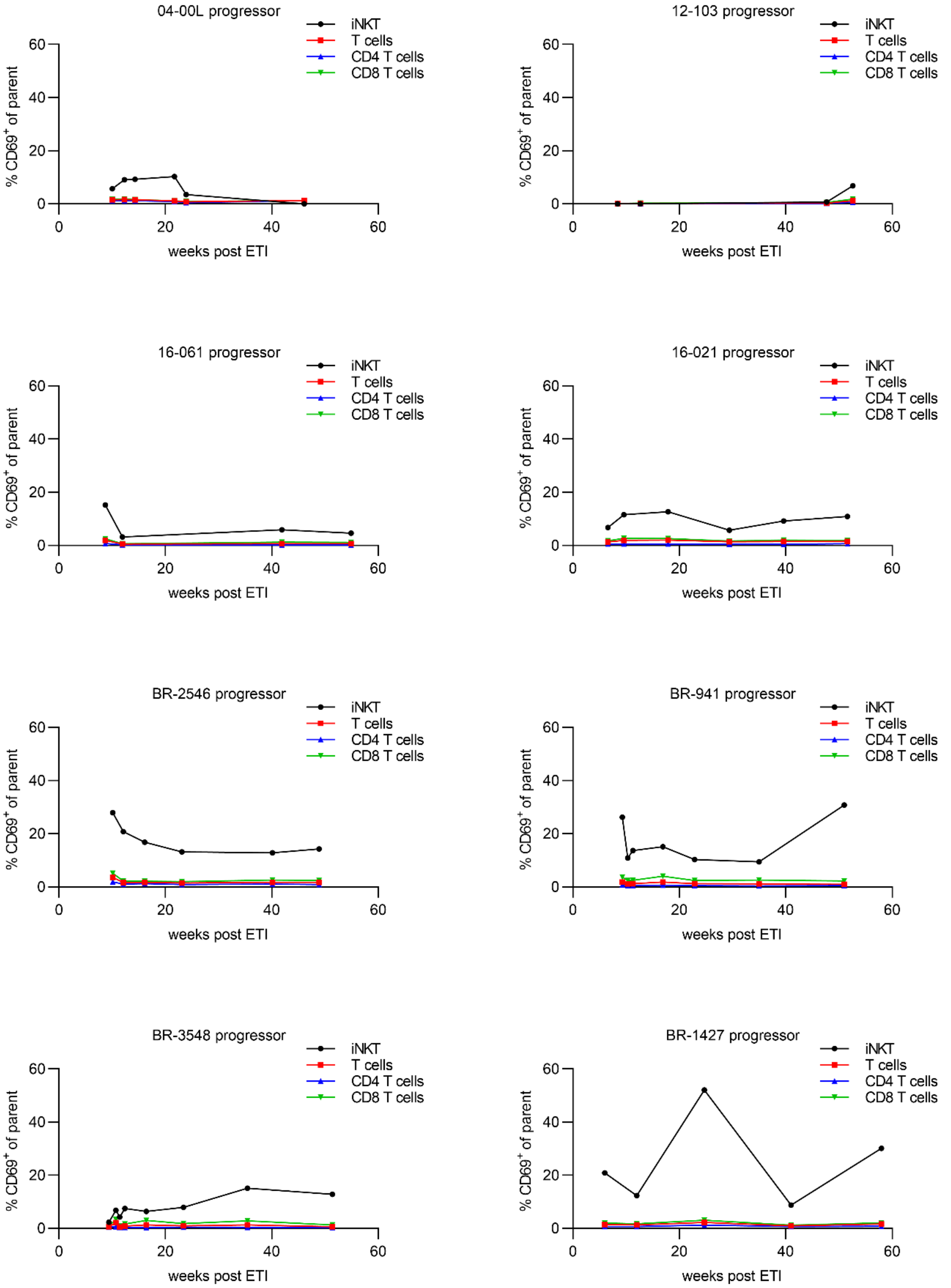

Supplementary Fig. 2. Frequency of activated iNKT and conventional T cells.

Frequency of CD38+ in resolvers (A) and progressors (B) and CD69+ in resolvers (C) and progressors (D) iNKT cells as wells as CD4+ and CD8+ conventional T cells

during the first year post ETI is shown. Patients with samples available from four or more time points are shown.

A

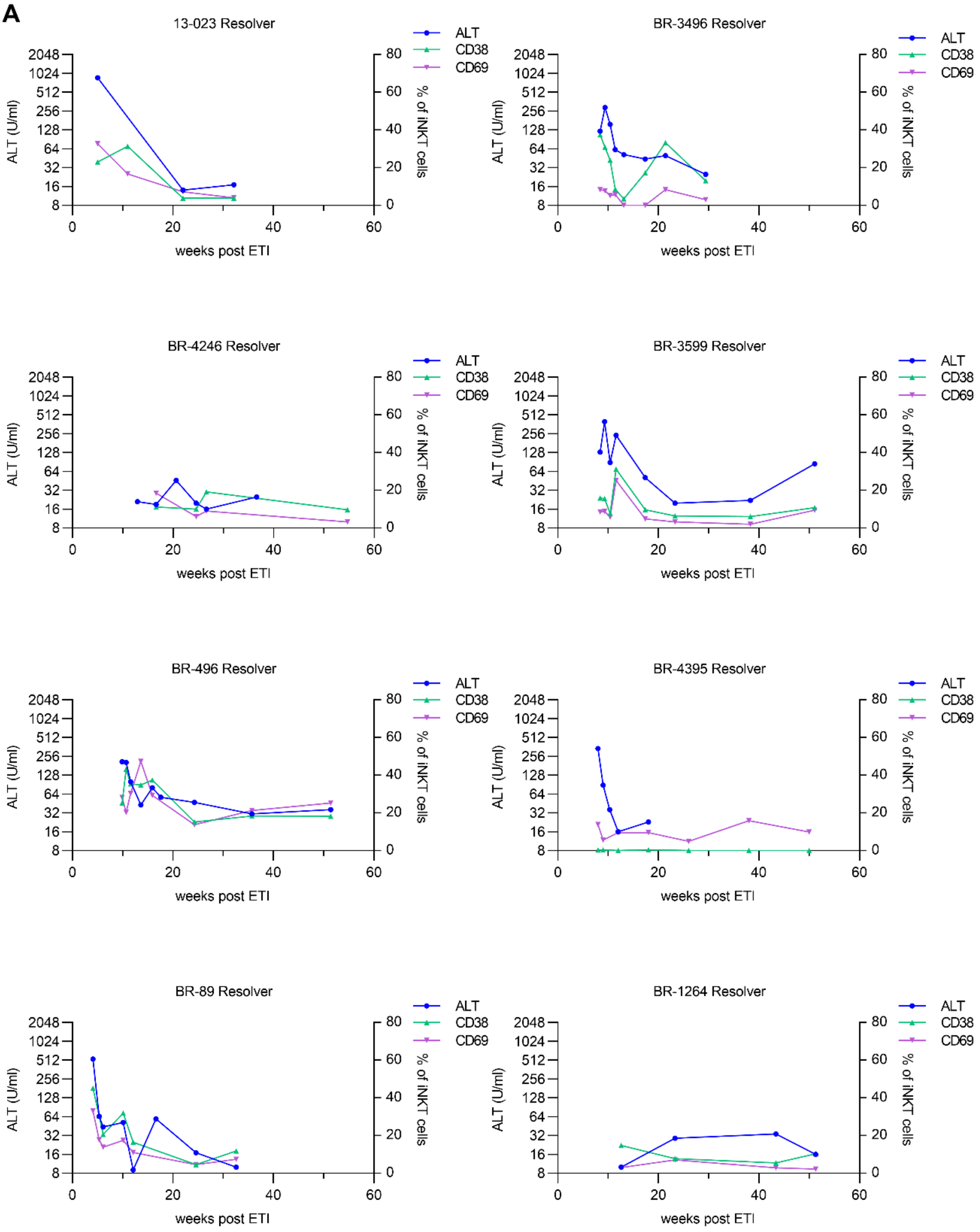
13-023 Resolver
BR-3496 Resolver
BR-4246 Resolver
BR-3599 Resolver
BR-496 Resolver
BR-4395 Resolver
BR-89 Resolver
BR-1264 Resolver
ALT
CD38
CD69
ALT (U/ml)
% of iNKT cells
weeks post ETI
2048
1024
512
256
128
64
32
16
8
0
20
40
60
80

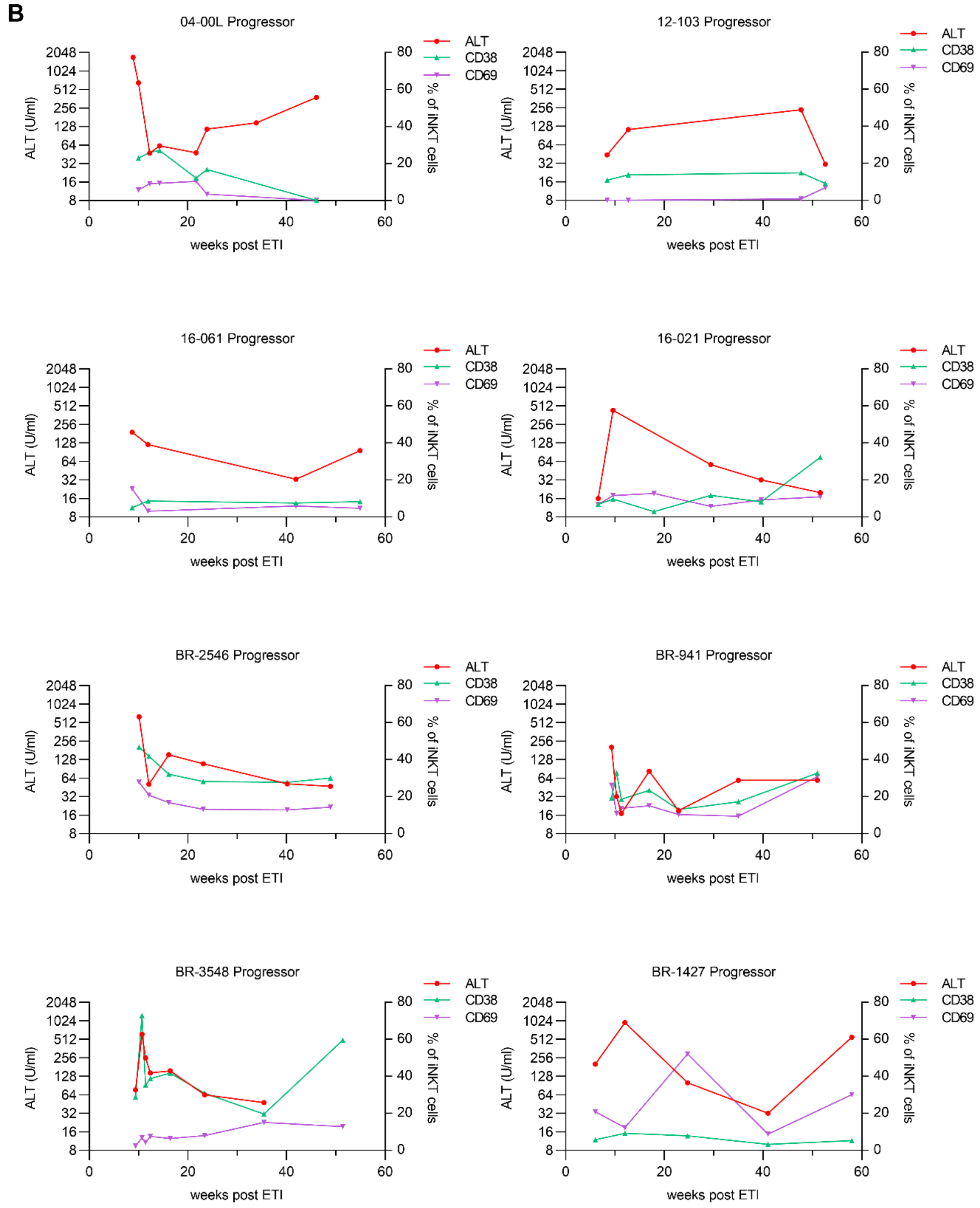

Supplementary Fig. 3. Invariant NKT cell activation and serum ALT levels in individual patients. Frequency of $CD38^+$ (green) and $CD69^+$ (purple) iNKT cells during the first year post ETI is shown. Blue (A, resolvers) or red (B, progressors) circles depict ALT levels. Exemplary patients that had the highest number of longitudinal samples with known ALT levels are shown.

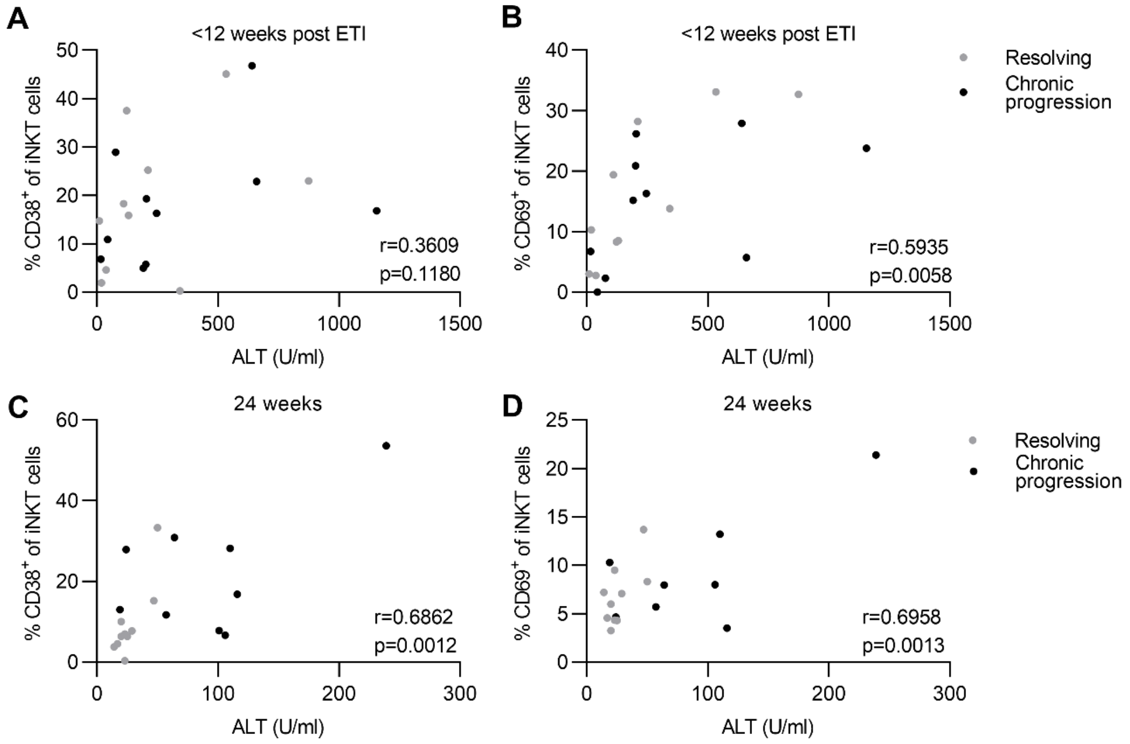

Supplementary Fig. 4. Invariant NKT cell activation and serum ALT levels at individual time points. (A + B) Correlation of ALT levels with the frequency of $CD38^+$ (A) and $CD69^+$ (B) iNKT cells is shown for the earliest available sample with known ALT levels that was sampled prior to 12 weeks post ETI (n = 20). (C + D) Correlation of ALT levels with the frequency of $CD38^+$ (C) and $CD69^+$ (D) iNKT cells is shown 6 months post ETI (16 to 32 weeks, n = 19). Each dot represents an individual patient. Resolvers and progressors are depicted by gray and black circles, respectively. Pearson correlation coefficient was used.

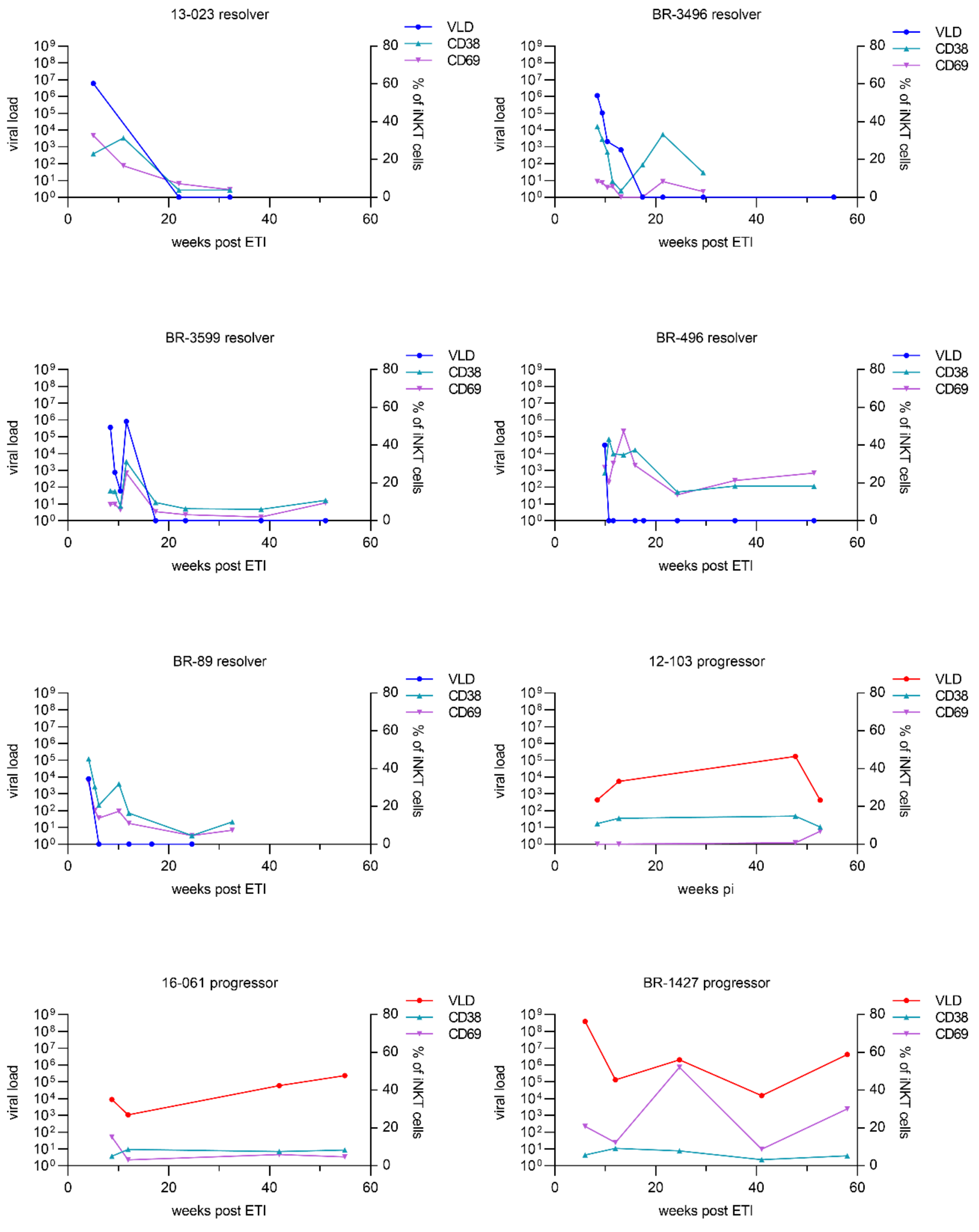

Supplementary Fig. 5. Invariant NKT cell activation and viral load. Frequency of CD38+ (green) and CD69+ (purple) iNKT cells during the first year post ETI is shown. Blue (resolvers) or red (progressors) circles depict viral load. Exemplary patients that had the highest number of longitudinal samples with analysed viral load are shown.

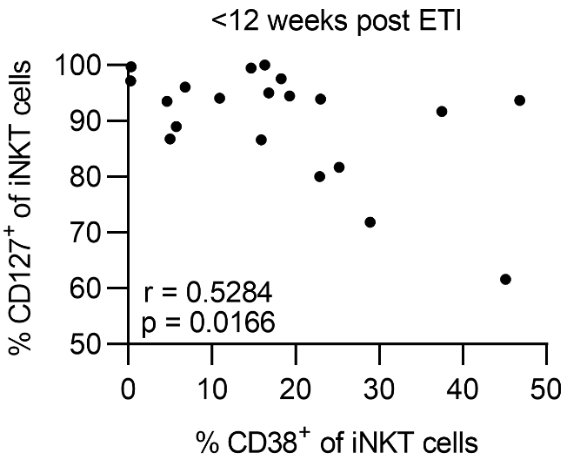

Supplementary Fig. 6. Frequency of CD38$^+$ and CD127$^+$ iNKT cells during early acute HCV infection. PBMC samples from the acute phase of HCV infection were analyzed by flow cytometry (n = 20). Expression of CD38 and CD127 was determined at the earliest available time point prior to 12 weeks post ETI and their correlation analysed by Pearson correlation analysis. Samples with less than 20 iNKT cells were excluded from the analysis.

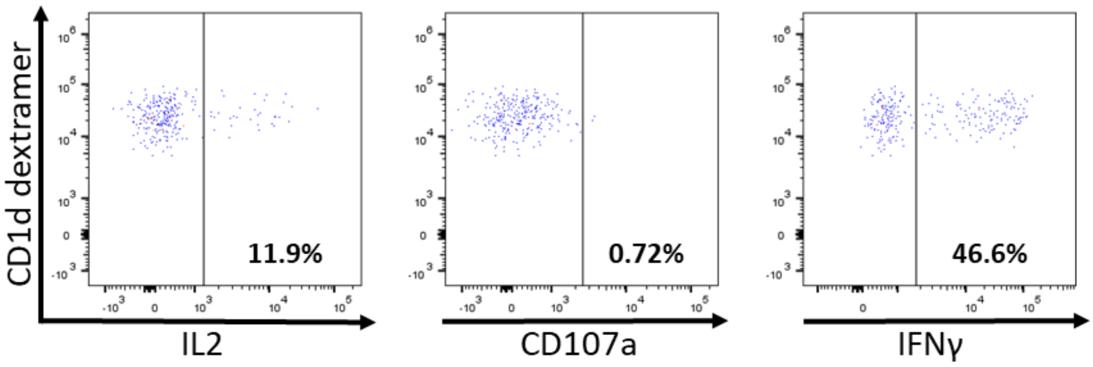

Supplementary Fig. 7. Intracellular cytokine staining of iNKT cell ex vivo.

Lymphocytes were identified and dead cells, doublets, and CD19^{+} cells, due to the unspecific binding of the CD1d dextramer to B-cells, were excluded. iNKT cells were defined as CD3^{+} and αGalCer loaded CD1d dextramer^{+}. Exemplary IFNγ, IL-2 production and CD107a expression by iNKT cells is shown.

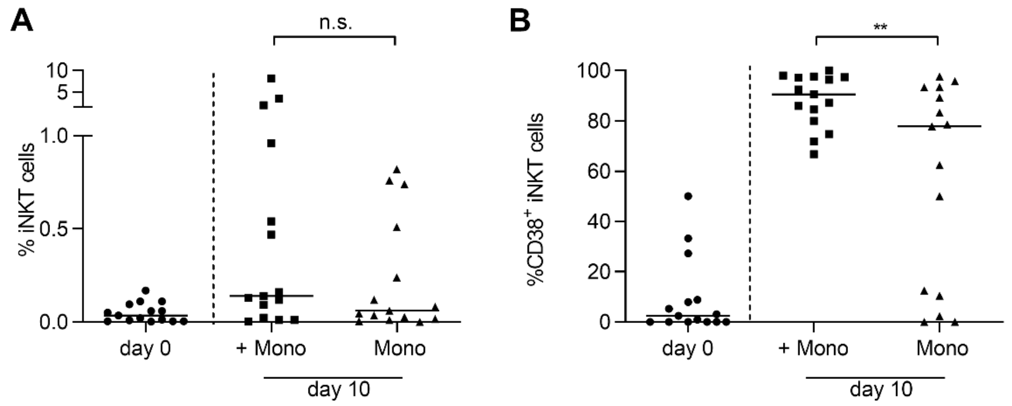

Supplementary Fig. 8. Depletion of CD1d results in impaired iNKT activation. Frequency (A) and $CD38^+$ expression (B) of iNKT cells ex vivo and after 10d of in vitro expansion with αGalCer and IL-2 from whole or monocyte depleted PBMCs is shown (n=15, Wilcoxon matched-pairs signed rank test, **>0.01). Monocytes were depleted by adherence to the plastic surface of the cell culture vessel for 3h at 37°C in R10. Suspension cells were collected without disturbing the adherent cells and gated like described in the methods section. Invariant NKT cells were defined via αGalCer loaded CD1d Dextramer staining.

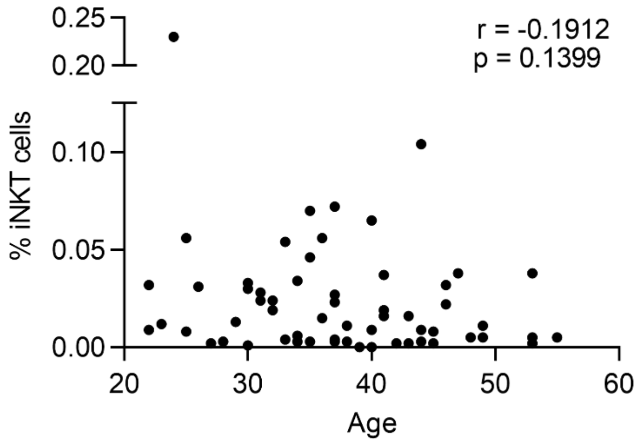

Supplementary Fig. 9. Association between the frequency of iNKT cells and patient age in PWID cohort. All patients independent of infection status were included in the analysis. Age in years and iNKT cell frequency of all $CD3^+$ lymphocytes is shown. Correlation was calculated by Spearman correlation analysis (n=61).

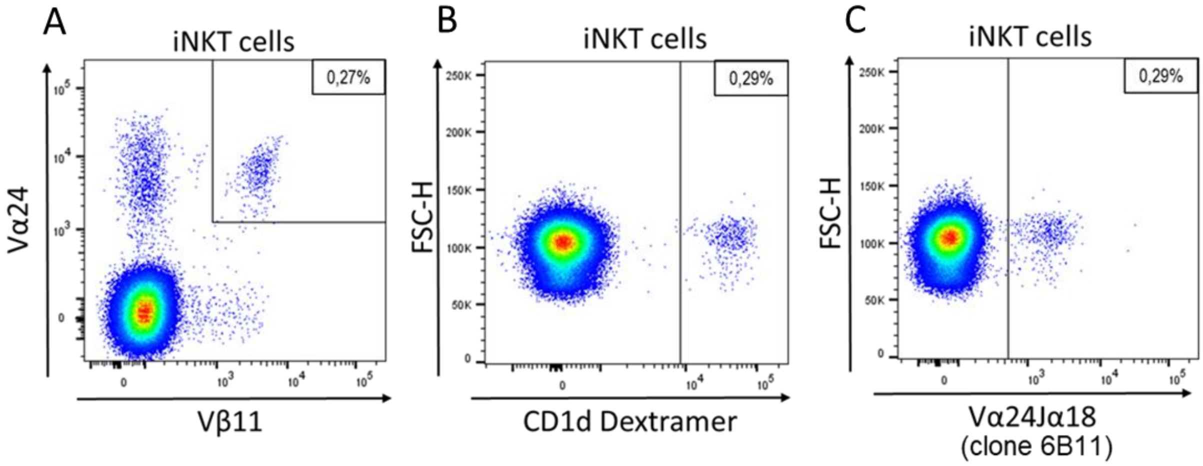

Supplementary Fig. 10. Comparison of iNKT staining reagents. iNKT cells were stained with (A) Vα24 and Vβ11, (B) αGalCer loaded CD1d Dextramer or (C) Vα24Jα18 antibody. PBMCs of healthy donors were stained like described in the methods section and gated on viable $CD3^+$ lymphocytes.
